# Integrating Transmission-Based Color Measurement with Clustering for Objective Grading of Translucent Plastics

**DOI:** 10.3390/s26216754

**Published:** 2026-10-25

**Authors:** Yueh-Feng Liu

**Affiliations:** General Education Center, Ming Chi University of Technology, 84 Gungjuan Rd., Taishan Dist., New Taipei City 243303, Taiwan; yfliu@mail.mcut.edu.tw

**Keywords:** K-means clustering, spectral clustering, BIRCH, GMM, color grading, color difference, Welch’s ANOVA, translucent plastic, quality control

## Abstract

Accurate and objective color grading is essential for quality control in the plastics industry. However, conventional visual inspection is subjective, while commercial color measurement instruments may be limited by measurement geometry, sample dimensions, or insufficient sensitivity to subtle color variations. This study proposes a low-cost transmission-based color measurement device integrating with unsupervised clustering for detecting subtle color differences in large-size and translucent general-purpose polystyrene samples. The system measures transmitted light through the samples and converts the sensor outputs into CIE L*a*b* color coordinates and derived color characteristics. A dataset comprising 128 GP samples classified into three quality grades by visual inspection is subsequently analyzed using Welch’s ANOVA and the Games–Howell post hoc test. To establish more objective grading criteria, K-Means, spectral clustering, Balanced Iterative Reducing and Clustering using Hierarchies (BIRCH), and Gaussian mixture models, which allow the number of clusters to be specified, are employed to determine quantitative color difference thresholds for the different grades. Jenks natural breaks, a simple one-dimensional classification method, is also used for comparison. Experimental results demonstrate that the proposed device has good measurement reproducibility and greater separation of subtle color variations than a commercial reflectance colorimeter. For the same set of GP samples, the color difference values measured using the proposed device range from 0.57 ± 0.08 to 3.06 ± 0.31, compared with 0.25 ± 0.04 to 0.70 ± 0.08 using the commercial colorimeter. BIRCH achieves the highest adjusted Rand index (0.50) and normalized mutual information (0.35), whereas K-Means produced the highest silhouette score (0.57). Overall, the color difference cutoff thresholds derived from BIRCH clustering show the closest agreement with the manufacturer’s practical quality control requirements. These results demonstrate that combining transmission-based color measurement with unsupervised machine learning provides an effective approach to objective and quantitative color grading of translucent plastic products. The proposed system provides a practical foundation for AI-assisted quality assurance and smart manufacturing in the plastics industry.

## 1. Introduction

In the modern polymer industry, color is not merely a sensory attribute of an object but also a critical factor in product quality, functionality, and brand identity. The wide range of applications for plastic products has created a growing demand for accurate, consistent, and reproducible color control. The problem addressed in this study originates directly from an industrial application. A plastic manufacturer produces general-purpose polystyrene (GP), a polymer synthesized through the free-radical polymerization of styrene monomers. GP is characterized by its transparency, good water resistance, ease of processing, and excellent colorability. During production, dyes of different colors and concentrations are incorporated into the polymer to manufacture products with various color specifications. At the final inspection stage, color quality control is performed to verify that the products conform to predefined specifications and meet customer requirements.

However, achieving reliable color control remains challenging from both scientific and technical perspectives. This difficulty arises primarily from the fundamental nature of color itself. Rather than being an intrinsic property of an object, color is a subjective visual perception resulting from a complex neurophysiological response to the interaction among the light source, the physical and chemical properties of the object, and the human visual system [1,2].

**Figure 1:**
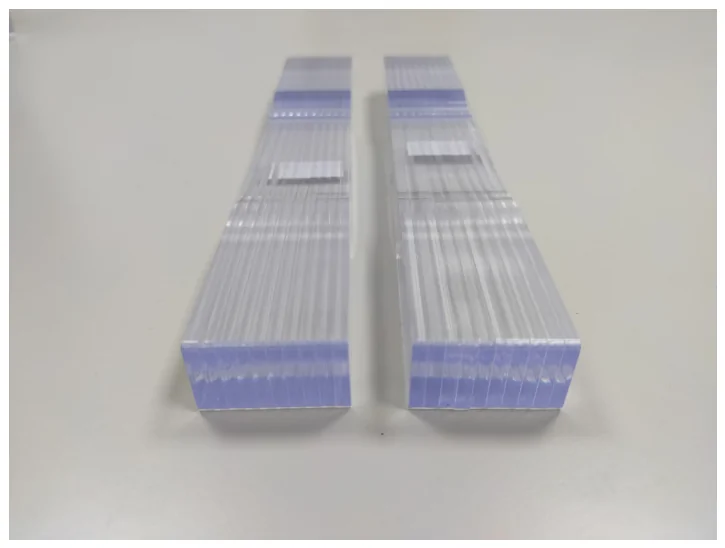
Two GP samples with a 21.5 cm length, 3.2 cm width, and 1.9 cm height. Color is only shown on 3.2 cm × 1.9 cm rectangular faces.

The GP samples in this study are rectangular cuboids with dimensions of 21.5 cm × 3.2 cm × 1.9 cm. Their color can be observed through the 3.2 cm × 1.9 cm rectangular faces, while the other four faces are transparent. Figure 1 shows a photograph of the GP samples.

The conventional method used in the industry to evaluate the color of GP samples is visual inspection. During the inspection process, the GP sample under evaluation and a reference sample with the standard color are placed side by side in a D65 color chamber [3,4]. An experienced inspector then visually compares the two samples to assess their degree of color agreement. If the color of the produced GP sample closely matches that of the reference sample, it is classified as grade 1. If a slight color deviation is observed, the sample is classified as grade 2. Finally, if the color deviation from the reference sample is more pronounced, the sample is classified as grade 3.

The visual inspection method has several inherent limitations [5]. First, differences in visual perception among inspectors may lead to subjective judgements and inconsistent quality control results. Second, the grading criteria lack objective and quantitative definitions, resulting in unclear boundaries between different GP sample grades. Third, the grading results are primarily categorical labels rather than quantitative measurements, which limits their direct integration into digital manufacturing systems. Therefore, establishing objective and digitized grading criteria is essential for smart manufacturing, data-driven quality control, and further applications of artificial intelligence.

The industry has attempted to use commercial colorimeters for color quality control and classification. Colorimeters meet the color measurement requirements of various fields such as the food industry [6,7]. However, conventional colorimeters generally lack sufficient discrimination to reliably distinguish among different color grades of translucent GP samples with the same specification. This limitation may be attributed to factors such as interference from surrounding ambient light. Spectrophotometers have also been considered for more precise color measurement. However, commercially available instruments are not suitable for the GP samples because of their relatively large dimensions. Therefore, there is a practical industrial need for a low-cost, effective, and sufficiently sensitive device capable of discriminating subtle color differences among plastic products.

In this study, we propose a transmission-based color measurement system capable of detecting subtle color variations in large, translucent GP samples. The measured color difference values are further analyzed and optimized using artificial intelligence (AI) techniques. The proposed method provides better color discrimination than reflective colorimeters and overcomes the sample size limitations associated with conventional colorimeters and spectrophotometers. The clustering provides an objective approach for establishing quantitative color-grading thresholds. Its low cost represents an additional advantage. The proposed method addresses the limitations of conventional color-grading approaches currently used in the plastics industry by providing objective and quantitative color evaluation. This work provides a practical foundation for AI-assisted quality assurance and the advancement of smart manufacturing in the plastics industry.

The remainder of this paper is organized as follows. Section 2 reviews existing color measurement methods and applications of unsupervised clustering. Section 3 describes the proposed device, statistical analysis methods, and clustering algorithms. Section 4 presents and discusses the experimental results. Finally, Section 5 summarizes the results and concludes the paper.

## 2. Related Works

### 2.1. Color Measurement

The development and adoption of instrumental color measurement techniques, such as spectrophotometers and colorimeters, have represented significant advances toward objective and reproducible color evaluation. These instruments provide quantitative and accurate color data, enabling more consistent and reliable quality control across different production batches.

Spectrophotometers measure spectral power, reflectance, and absorbance as functions of wavelength, providing a fundamental physical basis for instrumental color measurement. Spectrophotometry is therefore widely used in both industry and research for objective and quantitative color evaluation. However, color measurement of plastic materials presents particular challenges compared with that of opaque paints or textiles. Plastics are manufactured in various forms, including pellets, powders, films, sheets, and molded components, and may exhibit opaque, translucent, or transparent optical properties. Consequently, measurement results can be strongly influenced by factors such as measurement geometry, sample presentation, internal scattering and absorption, surface gloss and texture, and instrument design.

Recent reviews have highlighted the multifactorial complexity of color measurement in polymeric materials [8]. In particular, the effects of instrument geometry and surface characteristics on measured color have received considerable attention. Measurements of the same sample using different geometries or different conditions of specular-component inclusion or exclusion may produce different colorimetric results and perceived color differences. These effects are especially important for injection-molded plastics, where surface finish and internal light scattering interact to influence the apparent color.

Previous studies [9,10] have quantified the effects of measurement geometry and surface characteristics on measured color and proposed appropriate measurement strategies and correction models. For translucent plastics and thin films, subsurface scattering and multiple internal light interactions introduce additional complexity, causing the apparent color to depend strongly on sample thickness, substrate or backing conditions, and viewing geometry. Accordingly, research in this area has progressed from descriptive measurements toward modeling approaches capable of predicting perceived color under different thickness and backing conditions. Recent studies [11,12] have further extended these models and proposed measurement procedures for translucent polymer materials, including pellets and films.

Sample presentation is another important consideration in spectrophotometric color measurement because it can significantly affect measurement reproducibility. Pellets and powders, for example, exhibit light-scattering and packing effects that may produce color measurements different from those of molded components made from the same material. Previous studies [8,11] have documented measurement procedures and provided recommendations for sample preparation, presentation, and instrument selection according to the physical form and optical properties of polymer samples.

For manufacturing processes such as extrusion, molding, and compounding, online spectrophotometric systems capable of real-time measurement have been developed and applied to process monitoring and quality control [13]. More recent developments have focused on compact and robust sensing systems, their integration with automated process-control platforms, and algorithms capable of compensating for variations in surface and measurement conditions. Comparative studies have also investigated the trade-offs between conventional benchtop or portable spectrophotometers and lower-cost inline sensors for industrial process control [14].

Overall, spectrophotometry remains a preferred approach for accurate color measurement of plastic materials. However, its practical application may be constrained by measurement geometry, instrument configuration, cost, and sample dimensions, particularly when large shaped plastic products must be evaluated.

Colorimeters provide a more economical alternative for color specification, comparison, and quality control in various fields, including industrial manufacturing, imaging, and medicine. These instruments convert optical measurements into colorimetric coordinates associated with human color perception, such as the CIE XYZ and CIE L*a*b* color spaces, as well as various color difference and color appearance metrics [15,16,17]. Their outputs are typically expressed directly as tristimulus or derived colorimetric values that quantitatively characterize different dimensions of perceived color. Compared with spectrophotometers, colorimeters are generally more portable, less complex, more economical, and capable of rapid measurements. However, they do not provide full spectral information and typically offer less flexibility in the selection of illumination conditions and optical filters. Consequently, colorimeters may provide lower measurement accuracy and sensitivity than spectrophotometers, particularly when subtle color differences must be distinguished. In addition, most commercially available colorimeters operate in reflectance mode, whereas transmission-based colorimeters are generally designed for transparent liquid samples rather than solid materials.

In recent years, computer vision systems have emerged as powerful tools for color measurement, particularly in the quality control of nonhomogeneous materials or complex surfaces where pixel-level information is valuable. These systems capture digital images under controlled illumination and extract color features in standardized color spaces, such as RGB, HSV, and CIE L*a*b* color spaces [18,19,20,21,22]. Comparative studies have demonstrated that properly calibrated computer vision systems can achieve color-measurement performance comparable to that of spectrophotometers, particularly when optimized color-conversion models are employed [19,20]. These methods continue to advance through improvements in calibration procedures, machine learning techniques, and multispectral imaging.

Spectral imaging techniques, including hyperspectral imaging (HSI) and multispectral imaging (MSI), capture both spatial and spectral information, enabling more precise color measurement and material characterization than conventional RGB imaging [23]. Unlike standard cameras, which capture three broad color channels (*R*, *G*, and *B*), MSI acquires data over a limited number of discrete wavelength bands, whereas HSI records hundreds of narrow, contiguous spectral bands to form a high-resolution spectral data cube. Both techniques enable the reflectance spectrum at each pixel to be estimated and combined with standard color-matching functions to calculate perceptually relevant color coordinates and ΔE values under different illuminants. Because spectral imaging provides spatially resolved spectral information, it supports region-based analyses of color uniformity, spectral variation, and color–texture relationships that cannot be obtained using point-based instruments such as spectrophotometers. However, hyperspectral datasets are large and require advanced computational processing. Moreover, HSI and MSI systems are generally more expensive than conventional RGB cameras and basic colorimeters.

**Figure 2:**
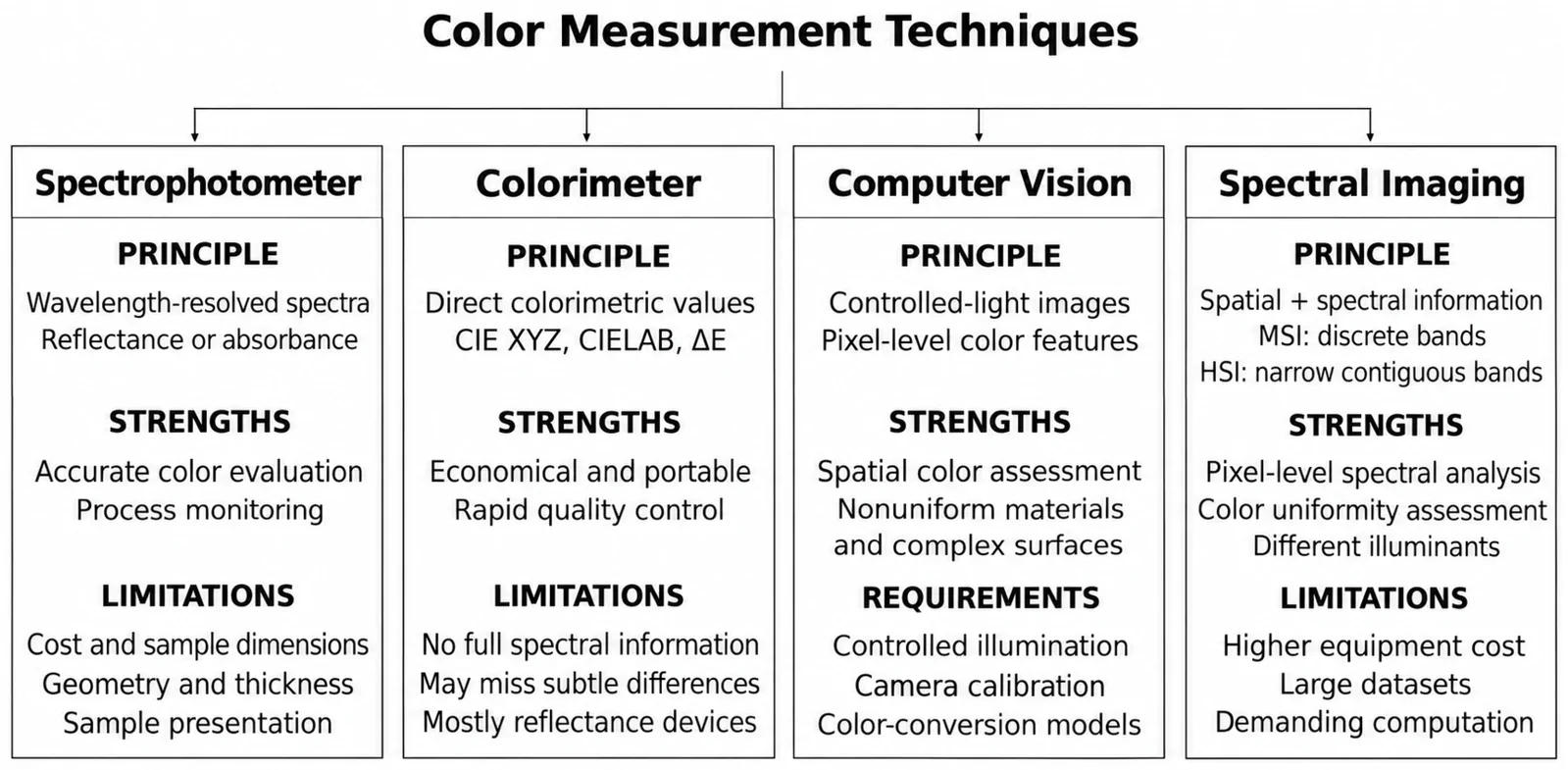
Summary of the color measurement techniques.

Deep learning-based color measurement is another promising research field. The study in [24] introduces an attention-based model for evaluating color differences in photographic images. Unlike conventional color difference formulas developed primarily for uniform color patches, the proposed method accounts for the spatial importance of local color variations and demonstrates stronger agreement with subjective evaluations. However, because the model is designed for photographic images, its application to industrial materials requires controlled illumination, camera calibration, and validation using application-specific samples. The study in [25] develops a deep learning-based metric for evaluating color differences in complex photographic images. Unlike CIE L*a*b*-based formulas, which are primarily developed from experiments involving uniform color patches, the proposed method jointly considers color information and spatial structure. Trained using human perceptual judgements, the model demonstrates stronger agreement with observers when evaluated on a large-scale dataset. Figure 2 summarizes color measurement techniques.

### 2.2. Unsupervised Machine Learning

Unsupervised machine learning clustering is an important data analysis technique for discovering latent structures and patterns within unlabeled datasets. The fundamental objective of clustering is to partition data instances into homogeneous groups such that data points within the same cluster exhibit high similarity, whereas those belonging to different clusters exhibit greater dissimilarity. Unlike supervised learning, which relies on labeled training data to learn a mapping between input features and predefined target classes, unsupervised clustering operates without known ground-truth labels. Instead, it identifies groups based on the intrinsic characteristics of the data, such as their structure, distribution, topology, similarity, and density [26,27,28].

Based on their underlying principles, unsupervised clustering algorithms can be broadly classified into several major families, including partition-based, hierarchical, density-based, grid-based, and model-based approaches. Over time, research in clustering has focused on several important challenges, including improving computational scalability for large and high-dimensional datasets, identifying clusters with complex and non-globular structures, enhancing robustness to noise and outliers, and developing reliable methods for cluster validation and interpretation. More recent developments have also incorporated advanced machine learning and representation learning techniques to improve clustering performance for increasingly complex datasets [26,27,28,29,30].

The development of unsupervised clustering algorithms has facilitated technological advances and data-driven applications across a wide range of fields. The study in [31] investigates optimized K-Means color quantization across machine-based and perception-based color spaces. Experiments on 148 diverse digital images compare RGB, CIE XYZ, CIELUV, and CIEHCL at four quantization levels, with visual information fidelity used to assess image quality. RGB performs best for approximately half of the images, whereas CIE XYZ generally improves performance at higher cluster numbers. The results show that optimal color-space selection depends on image hue, chromaticity, luminance, and quantization level. Another study employs unsupervised dimensionality reduction and machine learning methods to identify polymer configurations and phase transitions [32]. It demonstrates that meaningful polymer states can be discovered without manually constructed order parameters and supports using unsupervised learning to reveal hidden structures within polymer data. The study in [33] introduces a fully unsupervised machine learning framework for detecting and clustering structural defects in atomic-resolution images. In the automotive industry, an unsupervised clustering framework incorporating enhanced feature engineering has been developed for long-term battery fault warning using real-world electric vehicle data [34]. The study in [35] reviews applications of unsupervised clustering techniques in Alzheimer’s disease (AD) research. Because AD is a complex and heterogeneous disease, clustering methods can help identify meaningful patterns and subgroups within patient and biomarker data that may not be readily revealed by conventional statistical analyses.

Although deep learning-based approaches have been increasingly adopted in areas such as computer vision and genomics, supply chain management (SCM) continues to rely largely on conventional and interpretable machine learning methods. Systematic reviews indicate that, despite the growing adoption of machine learning in response to increasing market complexity and volatility, K-Means and hierarchical clustering remain among the most widely used clustering techniques in SCM [36]. Typical applications include stock-keeping unit categorization, customer segmentation, and supplier risk profiling. One important reason for the continued use of classical clustering methods is their interpretability. For example, centroid-based distances can provide an intuitive explanation of why a supplier is assigned to a high-risk cluster, whereas clustering based on deep neural representations may be considerably more difficult to interpret.

**Figure 3:**
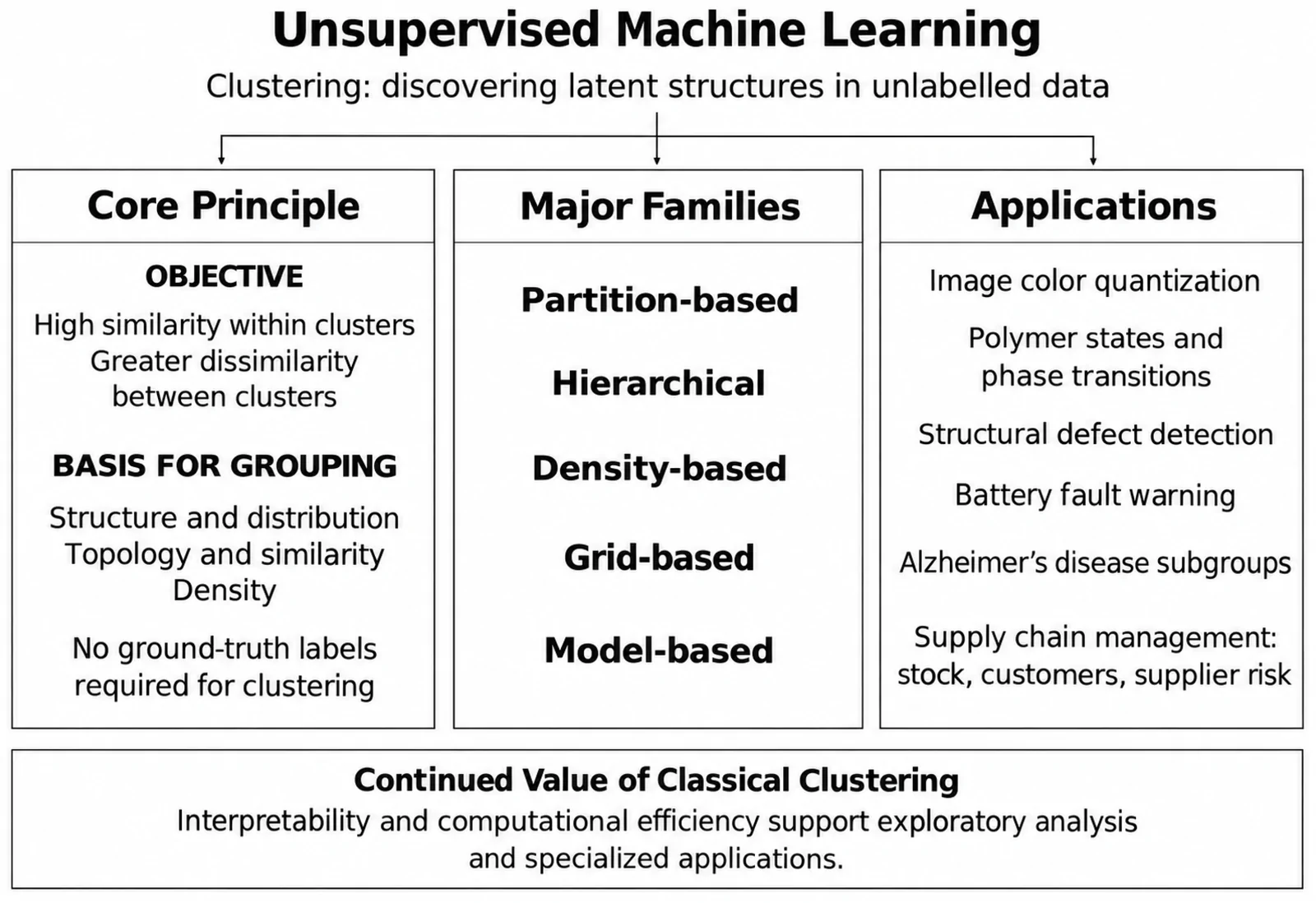
Summary of unsupervised machine learning.

These applications demonstrate that classical unsupervised machine learning clustering techniques remain essential for exploratory data analysis and specialized applications, particularly in domains where model interpretability and computational efficiency are important considerations. Figure 3 summarizes unsupervised machine learning.

## 3. Materials and Methods

### 3.1. Device

The device that we propose is composed of a 3D printing container, a color sensing module, a microcontroller board, a light-emitting diode (LED) light source, a liquid-crystal display (LCD) display, and a customized graphical user interface (GUI):

- The material of the container is made of acrylonitrile butadiene styrene (ABS) for achieving a higher resistance to corrosion by acids, alkalis, salts, and organic solvents [37,38]. The container has a cover that can completely cover the GP sample to be tested so that the color sensing module inside the container will not be affected by ambient light. The color of the container, including the cover, is black in order to minimize the effect of irrelevant light reflection.
- An LED is used as a light source whose color temperature is around 6500 K. The container has upper and lower decks connected on one side but not on the other. The GP sample to be tested is put on the upper deck, while the LED is set in the lower deck. The light emitted from the LED will first reflect from a white plate placed on the connected side of the decks and pass through the GP sample via the 3.2 cm × 1.9 cm rectangular face. The design of the container ensures that no light source passes through or reflects from the other four rectangular faces of the sample.
- A color sensing module (model: TCS34725) is put in the other side of 3.2 cm × 1.9 cm rectangular face to detect the light signal. The color sensing module has an infrared blocking filter integrated on-chip and localized to the color sensing photodiodes. This function will minimize the infrared spectral component of the incoming light and allow color measurements to be made accurately [39]. The color sensor provides a digital return concerning the sensing values of red, green, and blue, known as RGB. The specific RGB numerical values are able to be converted into the CIE L*a*b* color space [40]. The completion of this conversion process involves a series of necessary steps. Firstly, a normalization of the *R*, *G*, and *B* values must be done to ensure they are within a range of zero to one. This is achieved through the division of each value by 255.
  (1) $$R_{s}=\frac{R}{255},\;G_{s}=\frac{G}{255},\;B_{s}=\frac{B}{255}$$
  Subsequent to this normalization, we apply a non-linear correction to these new values, which is commonly called the gamma correction [41].
  (2) $$K=\left\{\begin{array}{cc} \frac{K_{s}}{12.92}, & K_{s}\leq 0.04045\\ {\left(\frac{K_{s}+0.055}{1.055}\right)}^{2.4}, & K_{s}>0.04045 \end{array}\right.$$
  where *K* is *R*, *G*, or *B*. Apart from this, it is required to utilize a specific matrix for the conversion of the gamma-corrected RGB values into the intermediate color space of CIE XYZ [41].
  (3) $$\left[\begin{array}{c} X\\ Y\\ Z \end{array}\right]=\left[\begin{array}{ccc} 0.4124564 & 0.3575761 & 0.1804375\\ 0.2126729 & 0.7151522 & 0.0721750\\ 0.0193339 & 0.1191920 & 0.9503041 \end{array}\right]\left[\begin{array}{c} R\\ G\\ B \end{array}\right]$$
  The next step concerns the normalization of the XYZ values to the reference white point.
  (4) $$x=\frac{X}{95.047},\;y=\frac{Y}{100.000},\;z=\frac{Z}{108.883}$$
  Finally, the CIE L*a*b* values are calculated from the XYZ values by using specific formulas to ensure perceptual uniformity [41].
  (5) $$f\left(t\right)=\left\{\begin{array}{cc} t^{\frac{1}{3}}, & t>{\left(\frac{6}{29}\right)}^{3}\\ \frac{1}{3}{\left(\frac{29}{6}\right)}^{2}t+\frac{4}{29}, & otherwise \end{array}\right.$$
  (6) $$L^{*}=116f\left(y\right)-16$$
  (7) $$a^{*}=500\left[f(x)-f(y)\right]$$
  (8) $$b^{*}=200\left[f(y)-f(z)\right]$$
  Upon completion of these conversions, three distinct values used to describe color visible to human eyes will be available. These are the lightness L*, the red–green axis value a*, and the yellow–blue axis value b*. The CIE L*a*b* color space can be further converted to chroma (C) using Equation (9),
  (9) $$C=\sqrt{{a^{*}}^{2}+{b^{*}}^{2}}$$
  and calculating hue (h) using Equation (10)
  (10) $$h=\arctan(\frac{b^{*}}{a^{*}})$$
  and converting the result to degrees. If we take the color measurements for both the GP sample with standard color and the one to be tested, a color difference ΔE [41,42]
  (11) $$\Delta E=\sqrt{{\Delta L^{*}}^{2}+{\Delta a^{*}}^{2}+{\Delta b^{*}}^{2}}$$
  quantity can be obtained [43], where
  (12) $$\Delta L^{*}=L_{standard}^{*}-L_{test}^{*}$$
  (13) $$\Delta a^{*}=a_{standard}^{*}-a_{test}^{*}$$
  (14) $$\Delta b^{*}=b_{standard}^{*}-b_{test}^{*}$$
  We try the most modern color difference formula $\Delta E_{00}^{*}$, which corrects for perceptual inconsistencies in the CIE L*a*b* space [16,17]. However, the simple color difference formula $\Delta E_{ab}^{*}$, i.e., Equation (11), has better performance in our particular case. We used the same standard reference sample to measure nine production samples with the same specifications as those in the dataset used in this study. Three samples are selected from each grade to represent the principal $\Delta E_{ab}^{*}$ range of 0.5 to 3.0. Table 1 lists nine samples with $\Delta E_{ab}^{*}$ ordered from small to large value and their corresponding $\Delta E_{00}^{*}$. The $\Delta E_{ab}^{*}$ is more sensitive to the color variations in the color region to be measured. Hence, the distinguishability of $\Delta E_{ab}^{*}$ for different grades is better than that of $\Delta E_{00}^{*}$. The following components are also included:
- Arduino Uno R3 is employed as a programmable microcontroller board to process the color sensing module signals, control the LED light source, and show the measured color results on an LCD display [44].
- A customized graphical user interface (GUI) is designed to take data, control the microcontroller board, and store data.

When the cover of the device is closed and no sample is placed inside the container, a calibration procedure can be performed. As light emitted from the LED reflects off the white plate placed inside the device, the color sensor will be calibrated to the white reference standard.

**Figure 4:**
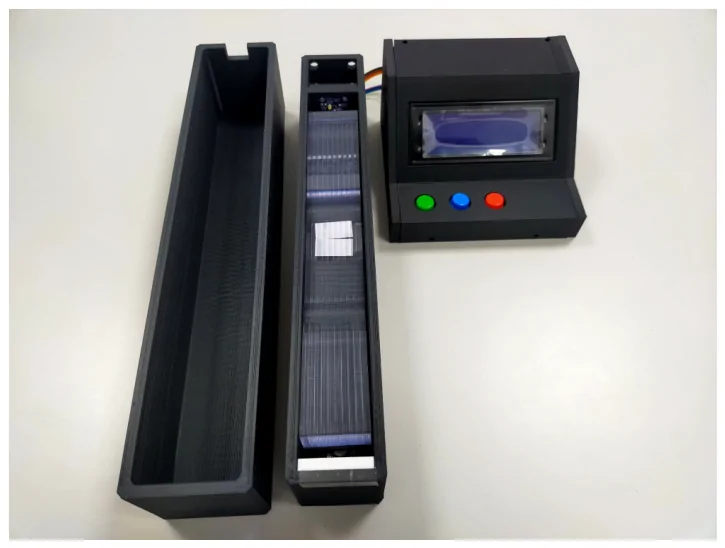
A photograph of our device: the left is the cover of the container, the middle is the container with a GP sample to be tested, and the right is a box with a microcontroller inside it and an LCD display mounted on it.

Figure 4 shows a photograph of the proposed device with all components fully assembled. The design of the device is based on transmission-mode color measurement. When light passes through a GP sample, it interacts with the dye molecules within the material. As the dye concentration increases, the transmitted light intensity decreases because of increased absorption. This effect becomes more pronounced as the optical path length through the sample increases. Therefore, a color-sensing module positioned on the opposite side of the sample can potentially detect subtle differences in light transmission among different color grades of GP samples.

Conventional commercial colorimeters may have difficulty distinguishing these subtle variations because most operate primarily in reflectance mode. Since GP samples of different color grades may contain only small differences in dye concentration, reflectance-based measurements may not provide sufficient sensitivity to discriminate among the grades. The proposed device therefore employs a transmission-based configuration to enhance discrimination of subtle variations in dye concentration while providing straightforward colorimetric quantities similar to those obtained from conventional colorimeters. Such quantitative outputs can be readily incorporated into subsequent data analysis and artificial intelligence applications. In this respect, the customized device combines the enhanced discrimination of transmission-based optical measurement with the simplicity and convenience of colorimetric evaluation.

### 3.2. Statistical Analysis Methods

The measured color data are first examined using statistical methods. Welch’s analysis of variance (Welch’s ANOVA) [45] is a robust alternative to conventional one-way ANOVA [46] for testing whether the means of two or more independent groups differ when the assumption of equal variances is violated. It is particularly suitable when the groups have unequal variances, unequal sample sizes, or both. Welch’s ANOVA can still be applied when the groups have equal variances and equal sample sizes, although it may be slightly less powerful than the conventional one-way ANOVA under these conditions. Nevertheless, its loss of efficiency is generally negligible [47]. Welch’s ANOVA assigns each group a weight based on its sample size and variance:

(15) $$w_{i}=\frac{n_{i}}{s_{i}^{2}}$$

where $n_{i}$ and $s_{i}^{2}$ are the sample size and variance of group *i*, respectively. The average of the group means ${\bar{X}}_{w}$ is defined as

(16) $${\bar{X}}_{w}=\frac{\sum_{i=1}^{k}w_{i}{\bar{X}}_{i}}{\sum_{i=1}^{k}w_{i}}$$

where *k* is the number of groups, and ${\bar{X}}_{i}$ is sample mean of group *i*. The Welch’s test statistic is defined as:

(17) $$F_{W}=\frac{\frac{1}{k-1}\sum_{i=1}^{k}w_{i}{\left({\bar{X}}_{i}-{\bar{X}}_{w}\right)}^{2}}{1+\frac{2(k-2)}{k^{2}-1}\sum_{i=1}^{k}\frac{{\left(1-w_{i}/\sum_{j=1}^{k}w_{j}\right)}^{2}}{n_{i}-1}}$$

The numerator degrees of freedom are

(18) $$df_{1}=k-1,$$

and the approximate denominator degrees of freedom are

(19) $$df_{2}=\frac{\;k^{2}-1\;}{3\sum_{i=1}^{k}\frac{{\left(1-w_{i}/\sum_{j=1}^{k}w_{j}\right)}^{2}}{n_{i}-1}}$$

A small *p*-value, commonly *p* < 0.05, indicates that at least one group mean differs significantly.

When Welch’s ANOVA indicated a significant difference among the three color grades, the Games–Howell post hoc test was performed for pairwise comparisons [48]. This procedure is selected because it does not require equal variances or equal sample sizes. The Games–Howell test statistic $q_{ij}$ is calculated as:

(20) $$q_{ij}=\frac{\sqrt{2}\left(\bar{x_{i}}-\bar{x_{j}}\right)}{\sqrt{\frac{s_{i}^{2}}{n_{i}}+\frac{s_{j}^{2}}{n_{j}}}}$$

where $s_{i}^{2}$ is the variance of group *i*, $s_{j}^{2}$ is the variance of group *j*, $n_{i}$ and $n_{j}$ are the corresponding group sizes, and $\bar{x_{i}}$ and $\bar{x_{j}}$ are means of the corresponding group.

The approximate degrees of freedom (df) are calculated using the Welch– Satterthwaite equation:

(21) $$\nu_{ij}=\frac{{\left(\frac{s_{i}^{2}}{n_{i}}+\frac{s_{j}^{2}}{n_{j}}\right)}^{2}}{\frac{{\left(s_{i}^{2}/n_{i}\right)}^{2}}{n_{i}-1}+\frac{{\left(s_{j}^{2}/n_{j}\right)}^{2}}{n_{j}-1}}$$

The Games–Howell test $q_{ij}$ statistic is evaluated using the studentized-range distribution with k (total number of groups) and the pair’s own degrees of freedom $\nu_{ij}$. The adjusted significance value *p* < 0.05 indicates that two group means are statistically significantly different.

**Figure 5:**
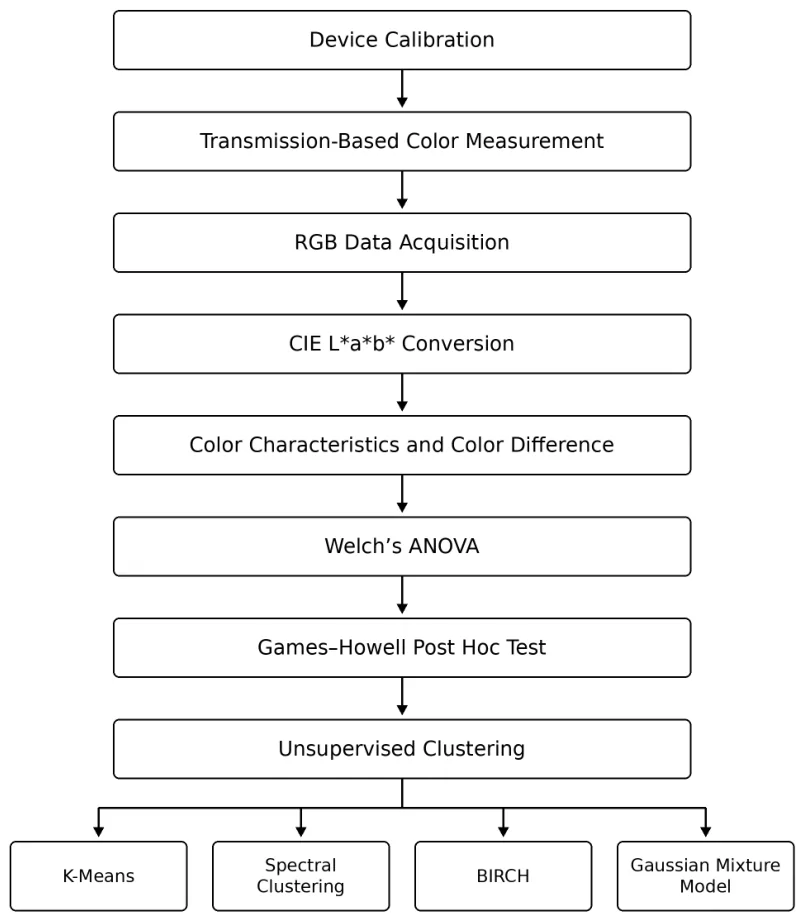
The inspection flow of analysis methods.

The inspection flow of analysis methods is presented in Figure 5.

### 3.3. Clustering Algorithms

After statistical analysis confirms that the color data measured by the proposed device can discriminate among different grades of GP samples, unsupervised machine learning methods are employed to further optimize the grading process. Partitioning methods are the most widely used among the major families of clustering algorithms. These methods aim to divide a dataset containing *n* data points into *k* mutually exclusive clusters, with each data point assigned to exactly one cluster. Partitioning algorithms generally seek to optimize an objective function defined by a specific measure of similarity or dissimilarity. K-Means is one of the most representative and widely adopted partitioning algorithms [49,50,51]. The standard K-Means algorithm operates iteratively by assigning each data point to the cluster with the nearest centroid and subsequently updating each centroid based on the mean of the data points assigned to that cluster. These two steps are repeated until convergence, with the objective of minimizing the within-cluster sum of squares (WCSS), thereby producing compact and well-separated clusters. The objective function *J* is defined as Equation (22),

(22) $$J=\sum_{i=1}^{k}\sum_{x_{j}\in C_{i}}{∥x_{j}-\mu_{i}∥}^{2}$$

where *k* is the number of clusters, $C_{i}$ is set of points in cluster *i*, $x_{j}$ represents a specific data point in cluster $C_{i}$, $\mu_{i}$ is centroid (mean) of cluster *i*, and $∥x_{j}-\mu_{i}∥$ is the Euclidean distance between point $x_{j}$ and the centroid $\mu_{i}$.

In the assignment step, each data point $x_{j}$ is assigned to the nearest centroid. The Euclidean distance in an n-dimensional feature space is determined by Equation (23),

(23) $$\mathrm{Euclidean}\;\mathrm{distance}\left(x_{j},\mu_{i}\right)=\sqrt{\sum_{d=1}^{n}{\left(x_{jd}-\mu_{id}\right)}^{2}}$$

where $x_{jd}$ is the d-th feature of data point $x_{j}$, and $\mu_{id}$ is the d-th feature of centroid $\mu_{i}$. Once all points are assigned, the positions of the *k* centroids are updated according to (24),

(24) $$\mu_{i}=\frac{1}{|C_{i}|}\sum_{x_{j}\in C_{i}}x_{j}$$

where $|C_{i}|$ is the total number of data points assigned to cluster $C_{i}$.

**Figure 6:**
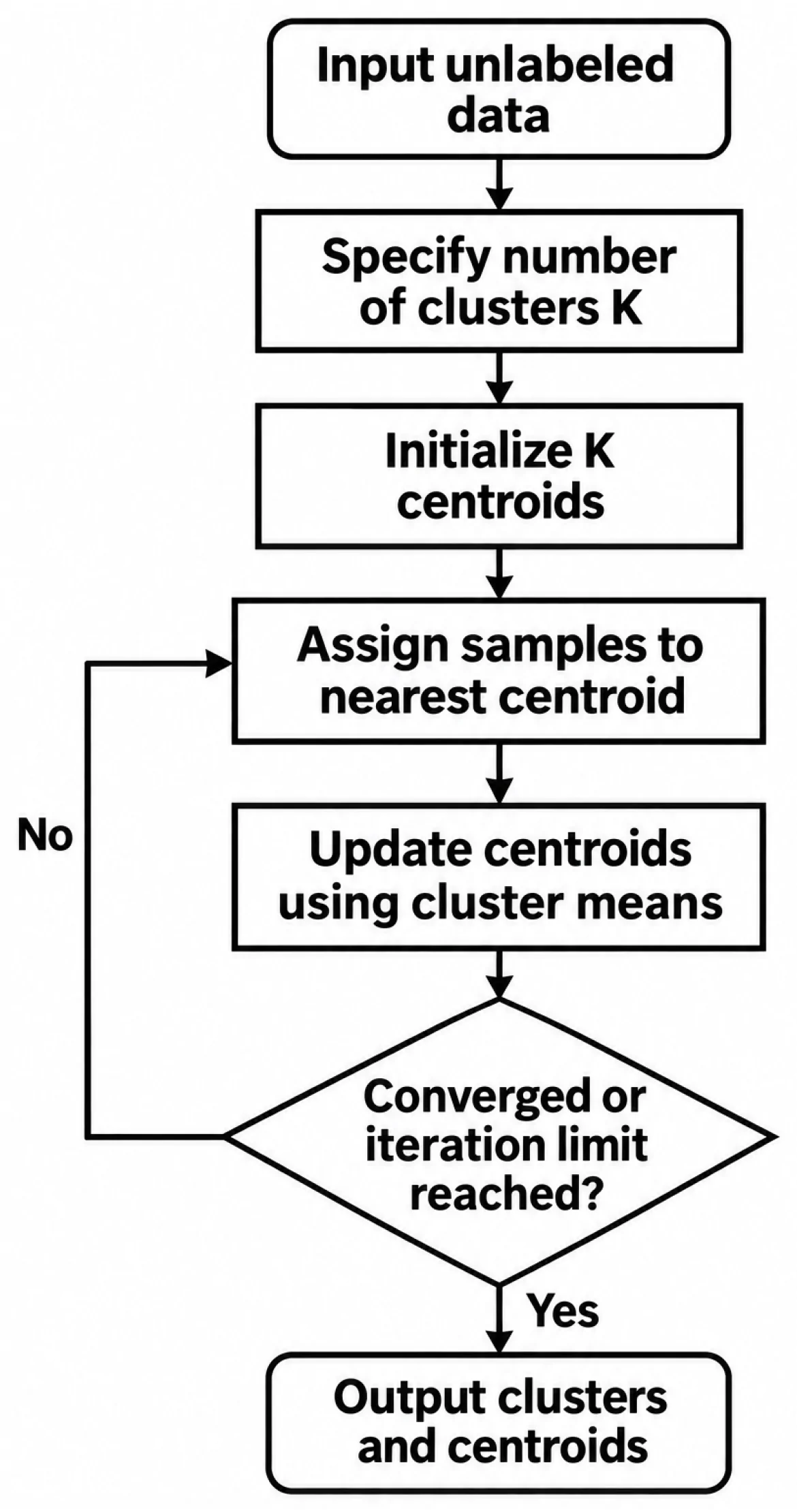
A flowchart of the K-Means clustering algorithm.

Although K-Means has a relatively low computational complexity that scales approximately linearly with the number of data points, it has several inherent limitations. It implicitly favors convex, approximately spherical, and isotropic clusters and therefore may perform poorly when the underlying data exhibit complex or non-convex structures, such as spirals or concentric circles. In addition, K-Means is sensitive to the initialization of cluster centroids, may converge to a local optimum, and requires the number of clusters *k* to be specified in advance. Figure 6 shows a flowchart of the K-Means clustering algorithm.

Spectral clustering is a graph-based unsupervised learning method that exploits the spectral properties of a similarity graph constructed from the data to identify intrinsic cluster structures [52]. Unlike conventional clustering approaches that rely primarily on geometric distances in the original feature space, spectral clustering maps data points into a lower-dimensional representation using the eigenvectors of a graph Laplacian matrix, where the underlying clusters may become more easily separable. The procedure typically begins with the construction of an affinity matrix, in which each element $W_{ij}$ represents the pairwise similarity between data points $x_{i}$ and $x_{j}$.

(25) $$W_{ij}=\exp\left(-\frac{∥x_{i}-x_{j}{∥}^{2}}{2\sigma^{2}}\right)$$

where σ is a parameter controlling the width of the Gaussian.

The degree matrix is calculated, which is a diagonal matrix where $D_{ii}$ is the sum of the similarities for the *i*-th data point.

(26) $$D_{ii}=\sum_{j=1}^{n}W_{ij}$$

The affinity matrix is then used to construct an unnormalized (27), symmetric normalized (28), or random-walk normalized (29) graph Laplacian.

(27) L=D−W

(28) $$L_{sym}=I-D^{-1/2}WD^{-1/2}$$

(29) $$L_{rw}=I-D^{-1}W$$

The eigenvalue problem for the graph Laplacian matrix is then solved to obtain the *k* smallest eigenvalues and their corresponding eigenvectors. The spectral embedding is constructed by collecting these eigenvectors, $U=[u_{1},u_{2},\ldots ,u_{k}]$, and stacking them as columns to form an n x k feature matrix *U*, as expressed in (30).

(30) $$U=\left[u_{1},u_{2},\ldots ,u_{k}\right]\in R^{n\times k}$$

**Figure 7:**
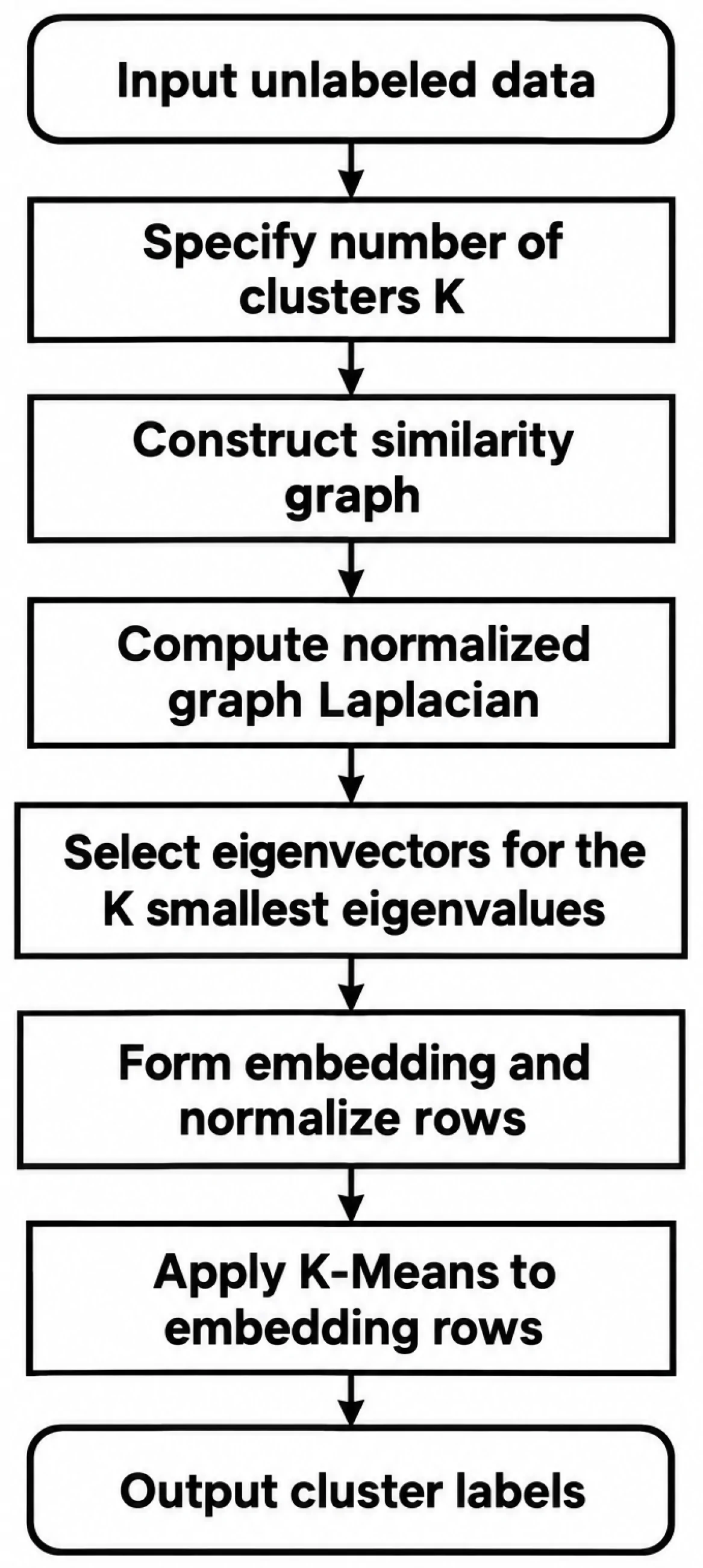
A flowchart of the spectral clustering algorithm.

Although spectral clustering is effective for identifying non-linear and non-convex cluster structures, its performance strongly depends on the appropriate construction of the similarity graph and the selection of relevant parameters. In addition, the computational cost associated with the eigen-decomposition of the graph Laplacian can be substantial, limiting its scalability to large datasets. Figure 7 shows a flowchart of the spectral clustering algorithm.

Balanced Iterative Reducing and Clustering using Hierarchies (BIRCH) [53,54,55] is an efficient clustering algorithm designed for large-scale datasets. It incrementally and dynamically organizes incoming data points into a compact hierarchical structure known as a Clustering Feature (CF) tree. The CF tree summarizes the dataset through sub-clusters, each represented by three statistical quantities: the number of data points, their linear sum, and their squared sum. For a cluster containing *N* d-dimensional data points $\left\{x_{1},x_{2},\ldots ,x_{N}\right\}$, the corresponding clustering feature can be defined as follows:

(31) $$CF=\left(N,\;LS,\;SS\right)$$

where LS is the linear sum (32) and SS is the squared sum of the points (33).

(32) $$LS=\sum_{i=1}^{N}x_{i}$$

(33) $$SS=\sum_{i=1}^{N}x_{i}^{2}$$

BIRCH calculates the metric *R*, which is the average distance from the member points to the centroid defined in Equation (34).

(34) $$R=\sqrt{\frac{\sum_{i=1}^{N}{\left(x_{i}-C\right)}^{2}}{N}}=\sqrt{\frac{SS}{N}-{\left(\frac{LS}{N}\right)}^{2}}$$

where *C* is the geometric center of the cluster expressed in Equation (35).

(35) $$C=\frac{LS}{N}$$

**Figure 8:**
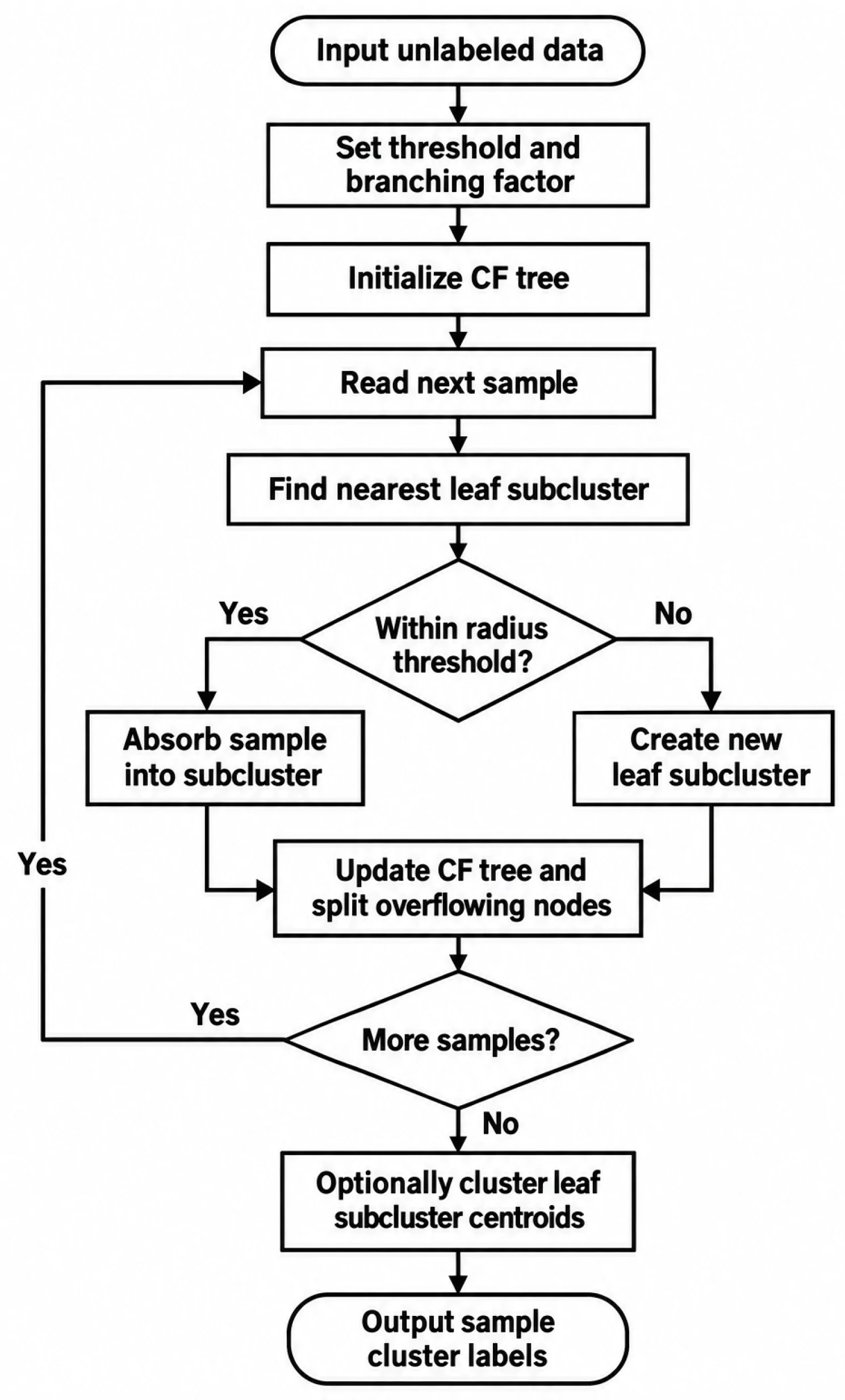
A flowchart of the BIRCH algorithm.

The CF tree is constructed incrementally by assigning each incoming data point to the nearest leaf entry, provided that the resulting sub-cluster radius *R* does not exceed a user-defined threshold. When this threshold is exceeded, the CF tree is updated through the creation or splitting of entries as necessary. In this manner, BIRCH efficiently compresses the original dataset while preserving its essential clustering characteristics. After the CF tree has been constructed, an optional global clustering algorithm, such as agglomerative clustering, can be applied to the leaf entries to further refine the final cluster assignments. Figure 8 shows a flowchart of the BIRCH algorithm.

Model-based clustering is a probabilistic approach to unsupervised learning that assumes the observed data are generated from a mixture of underlying probability distributions, with each distribution representing a latent cluster. Unlike distance-based or density-based methods, it explicitly defines a generative model and estimates cluster membership through statistical inference. A widely used example is the Gaussian mixture model (GMM), in which each cluster is represented by a Gaussian component characterized by its mean and covariance matrix, while the overall data distribution is expressed as a weighted combination of these components [56]. The probability density function for a single d-dimensional data point *x* in a GMM is expressed as (36),

(36) $$p\left(x\right)=\sum_{k=1}^{K}\pi_{k}N\left(x\;|\;\mu_{k},\Sigma_{k}\right)$$

where *K* number of Gaussian components; $\pi_{k}$ is a mixing coefficient, satisfying (37); $\mu_{k}$ is the mean vector of the *k*-th Gaussian; $\Sigma_{k}$ is the covariance matrix of the *k*-th Gaussian; and $N\left(x\;|\;\mu_{k},\Sigma_{k}\right)$ is equal to (38)

(37) $$\sum_{k=1}^{K}\pi_{k}=1,\;\pi_{k}\geq 0$$

(38) $$\frac{1}{{\left(2\pi \right)}^{d/2}{\left|\Sigma_{k}\right|}^{1/2}}\exp\left(-\frac{1}{2}{\left(x-\mu_{k}\right)}^{T}\Sigma_{k}^{-1}\left(x-\mu_{k}\right)\right)$$

where $|\Sigma_{k}|$ is the determinant of the covariance matrix, and $\Sigma_{k}^{-1}$ is its inverse.

Model parameters are commonly estimated using the expectation–maximization (EM) algorithm, which iteratively updates latent cluster assignments and model parameters to maximize the likelihood of the observed data [57]. This probabilistic framework enables soft clustering by estimating the posterior probability that each data point belongs to each cluster, thereby offering a more informative representation of membership uncertainty than hard-partitioning methods such as K-means.

**Figure 9:**
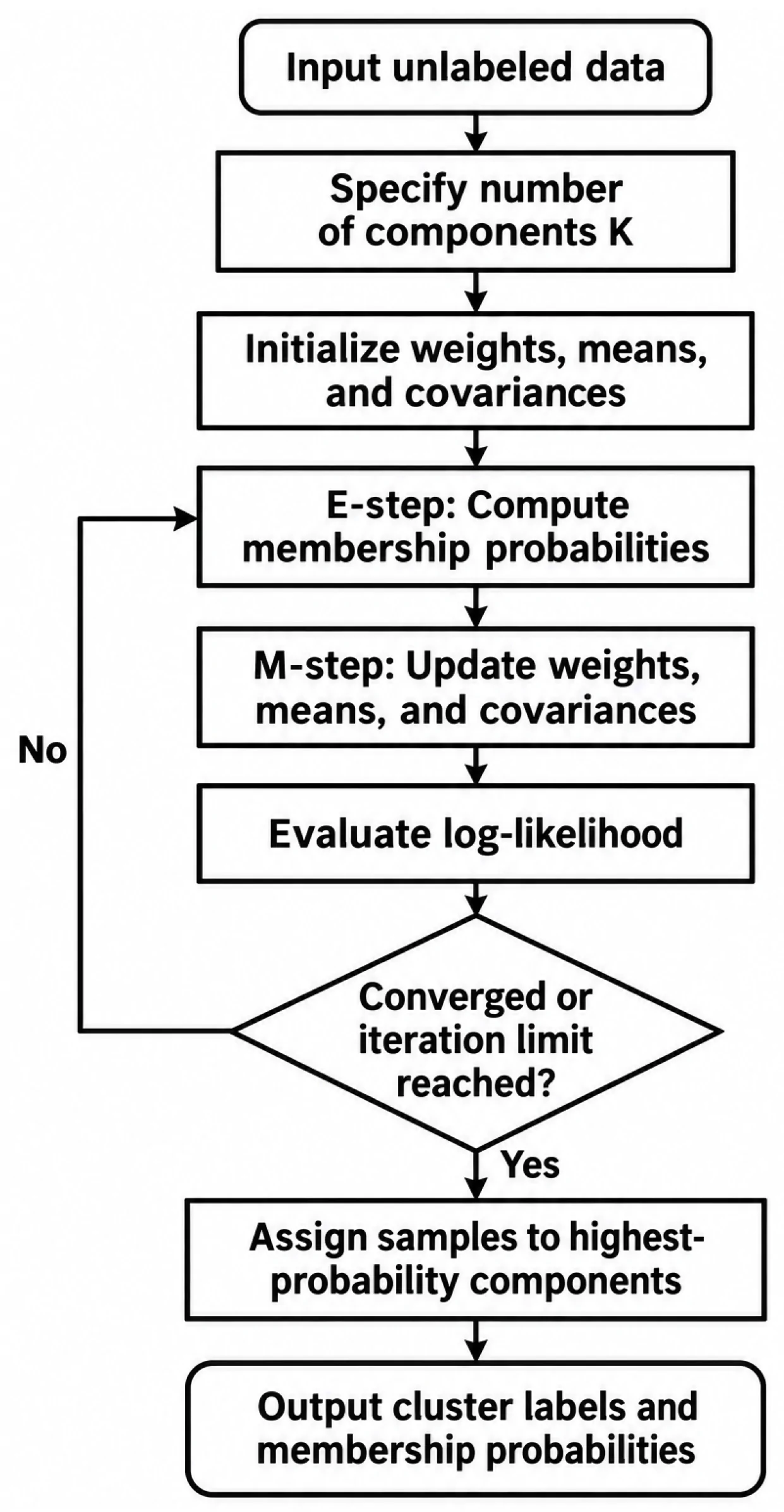
A flowchart of the Gaussian mixture model algorithm.

A key advantage of model-based clustering is its flexibility and interpretability, as different probability distributions and covariance structures can accommodate clusters with varying shapes, orientations, and volumes. Moreover, this approach can naturally handle missing data and be extended beyond Gaussian distributions to model a broad range of data structures. However, the performance of model-based clustering depends heavily on correctly specifying the underlying generative model, as model misspecification may produce inaccurate clustering results. The EM algorithm is also sensitive to parameter initialization and may converge to local optima, thereby reducing its stability for complex, multimodal datasets. Furthermore, the computational cost increases with the dimensionality of the data and the number of mixture components, limiting its scalability to large datasets. Figure 9 shows a flowchart of the Gaussian mixture model algorithm.

**Figure 10:**
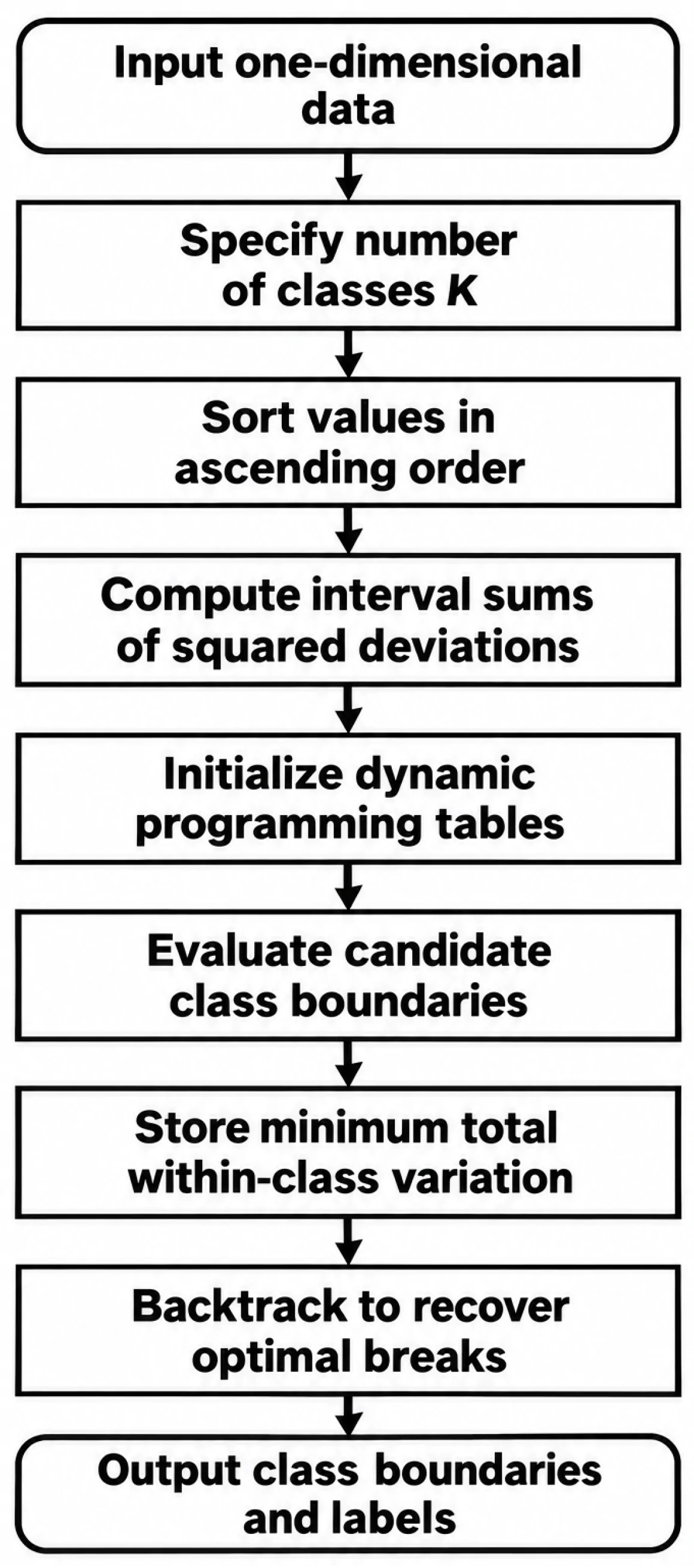
A flowchart of the Jenks natural breaks algorithm.

Jenks natural breaks is an unsupervised method that partitions one-dimensional numerical data into a specified number of groups [58]. It identifies boundaries that minimize variation within groups, producing classes whose values are relatively similar. For *n* observations divided into *K* groups, the objective is to minimize the within-class sum of squares (WCSS):

(39) $$\mathrm{WCSS}=\sum_{k=1}^{K}\sum_{x_{i}\in C_{k}}{\left(x_{i}-\mu_{k}\right)}^{2}$$

where $C_{k}$ is the *k*-th group and $\mu_{k}$ is its mean. Figure 10 shows a flowchart of the Jenks natural breaks algorithm.

## 4. Results and Discussion

The reproducibility of the proposed device is evaluated by repeatedly measuring the same GP sample over 10 consecutive days. Three measurements are performed each day at 8 h intervals, resulting in a total of 30 measurements. For each measurement, the sample is removed from the device and repositioned before measurement to account for potential variations associated with sample placement. In addition, each measurement is conducted 5 min after the device is turned on to ensure consistent operating conditions. The mean values, standard deviations, and coefficients of variation obtained from the 30 repeated measurements are summarized in Table 2.

The color measurement performance of the proposed device is compared with that of a commercial colorimeter (model: LS173) using the same set of GP samples representing different color grades and the same standard reference sample. The comparative results are presented in Table 3. In the proposed device, the GP sample is completely enclosed during measurement, and light is transmitted through the sample before reaching the color-sensing module. In contrast, when using the commercial colorimeter, the instrument is placed against one side of the sample, while the remaining sides are exposed to the surrounding environment. Moreover, the commercial colorimeter operates in reflectance mode by detecting light reflected from the sample surface.

The results demonstrate that the proposed device provides greater separation of subtle color variations among the GP samples. This improved discrimination can be attributed to the controlled illumination environment and the transmission-based measurement configuration, which enhances the interaction between light and the dye molecules within the sample. Based solely on the color difference value ΔE measured by the proposed device, the samples can be classified consistently with the visual inspection results. Accordingly, plastic manufacturers can establish quantitative ΔE thresholds for grades 1, 2, and 3 according to their specific quality control and commercial requirements. In contrast, the ΔE values obtained using the commercial colorimeter exhibit insufficient sensitivity to clearly distinguish among the different color grades, resulting in poorer discrimination than that achieved with the proposed device.

Data are collected over several months because GP samples of a given specification are not produced on a daily or weekly basis; instead, the production schedule is determined by customer demand. To maintain consistency in the quality control procedure, the color of each GP sample is measured at a predefined time point immediately after the sample becomes available. Before measuring new samples from each production batch, the same standard reference sample is measured to verify the stability of the measurement system. If the measured color values of the standard sample deviate from their mean values by more than one standard deviation, a calibration procedure is performed or the standard sample is replaced. In addition, the visual grade assigned to each sample by experienced inspectors is recorded for comparison. In this study, we present the results for the GP specification with the largest number of available samples, comprising a total of 128 samples.

**Figure 11:**
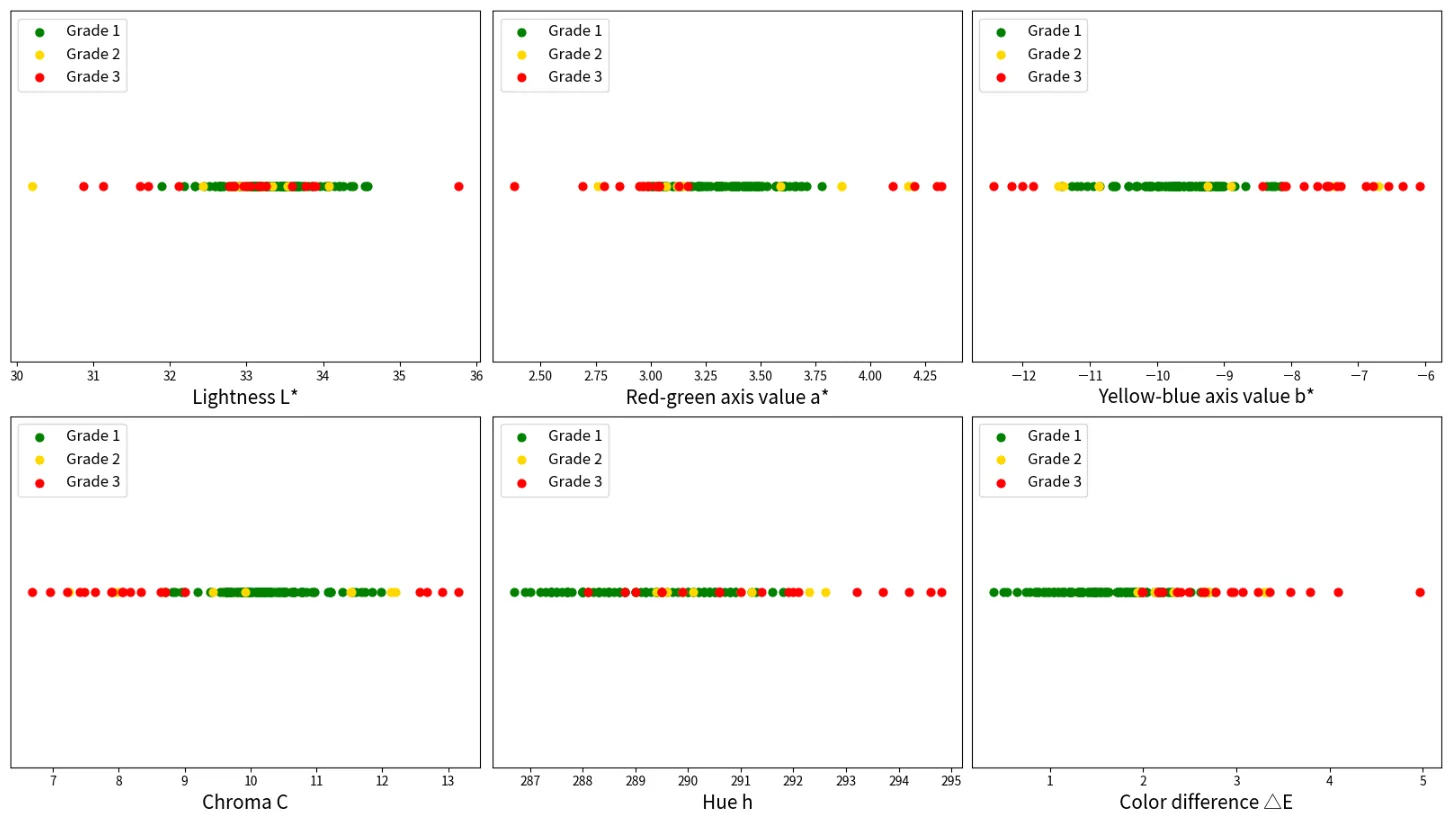
The results of the color measurement obtained from one specification of GP samples. (**Top left**): lightness L*, (**top middle**): red–green axis value a*, (**top right**): yellow–blue axis value b*, (**bottom left**): chroma C, (**bottom middle**): hue h, (**bottom right**): color difference ΔE. Green points: grade 1 samples, yellow points: grade 2 samples, and red points: grade 3 samples.

**Figure 12:**
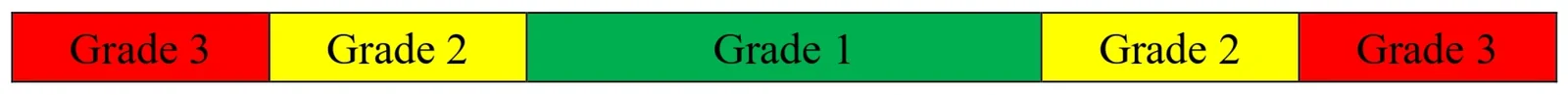
A schematic illustration depicting the ideal distribution of the characteristic values for color measurements, which is able to distinguish between different grades of samples. Note that the ranges of different grades in this illustration do not reflect the actual cases or refer to any distribution function. The schematic illustration shows the relative position for different grades.

**Figure 13:**
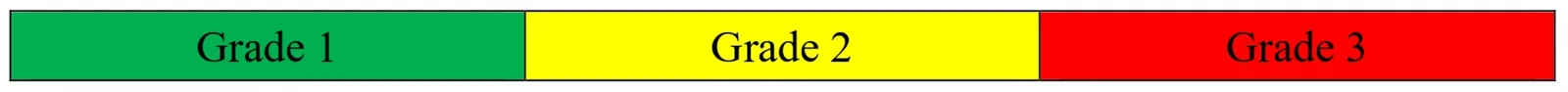
A schematic illustration demonstrating the ideal distribution of color difference used to differentiate various grades of samples. The ranges of different grades in this illustration do not reflect the actual cases or refer to any distribution function. The schematic illustration shows the relative position for different grades.

Figure 11 shows the distributions of the measured values for each color characteristic, together with the corresponding visual inspection grades. Green, yellow, and red data points represent grade 1, grade 2, and grade 3 GP samples, respectively. For a color characteristic to effectively distinguish among different grades, the distributions of the measured values are expected to follow a pattern similar to the schematic illustration in Figure 12. In Figure 11, the red–green axis value a*, yellow–blue axis value b*, and chroma C color characteristics exhibit a two-sided grading pattern, with grade 1 samples located within the central reference range. Grade 2 samples fall on either side of this range, whereas grade 3 samples occupy the outermost ranges, corresponding to larger deviations in either direction. The measured lightness L* and hue h characteristics do not show this two-sided grading pattern. The color difference, ΔE, is treated separately because its magnitude directly represents the degree of color deviation from the standard reference sample; therefore, its expected distribution follows the schematic pattern shown in Figure 13. In Figure 11, the color difference ΔE exhibits a one-sided grading pattern, with grade 1 occupying the lowest-value range, grade 2 the intermediate range, and grade 3 the highest-value range. Increasing the color difference therefore corresponds to a progressively greater deviation from the reference sample.

We check the ΔE noise-normalized separability on 128 samples. The separability between two grades is quantified using the absolute difference between their mean measured values divided by their pooled within-grade standard deviation [59]:

(40) $$S_{ij}=\frac{\left|{\bar{x}}_{i}-{\bar{x}}_{j}\right|}{s_{pooled}},\;s_{pooled}=\sqrt{\frac{\left(n_{i}-1\right)s_{i}^{2}+\left(n_{j}-1\right)s_{j}^{2}}{n_{i}+n_{j}-2}}$$

where ${\bar{x}}_{i}$ and ${\bar{x}}_{j}$ are the grade means, $s_{i}$ and $s_{j}$ are their standard deviations, and $n_{i}$ and $n_{j}$ are their sample sizes. This measure expresses the between-grade difference relative to the variability within the grades. Larger values indicate greater separation relative to within-grade variability, whereas values close to zero indicate similar grade means. The separability between different grades is presented in Table 4.

Although the distributions corresponding to different grades are not completely separated, the observed overlap may be partly attributed to the subjective nature of visual inspection, which is used to assign the reference grade labels. As discussed previously, differences in visual perception and judgement among inspectors may result in inconsistencies in the assigned color grades.

We perform Welch’s ANOVA test for each characteristic value of color measurement to check whether the means of different grades differ significantly. For each bidirectionally distributed color characteristic, including lightness L*, the red–green axis value a*, the yellow–blue axis value b*, chroma C, and the hue h, the data are divided into five groups. Grade 2 samples with values greater than the mean value of all grade 1 samples are labeled as “grade 2R”, whereas those with values less than or equal to the grade 1 mean value are labeled as “grade 2L”. Similarly, grade 3 samples with values greater than the grade 1 mean value are labeled as “grade 3R”, while those with values less than or equal to the grade 1 mean value are labeled as “grade 3L”. The labels of the grade 1 samples remain unchanged. Summaries and results of the Welch’s ANOVA and Games–Howell test for each measured color characteristic value are shown in Table 5, Table 6, Table 7, Table 8, Table 9 and Table 10. In these tables, group is a specific category or subset of measured color characteristic, count is the number of data points in each group, average is the mean value of data points in each group, variance is the average of the squared differences from the mean, F-statistic is the Welch’s test statistic, $df_{1}$, $df_{2}$ are degrees of freedom as described in Section 3.2. The *p*-value in Welch’s ANOVA is the probability of observing group means as different as (or more different than) the ones in the data points, assuming the null hypothesis is true (that all population means are equal). Statistic is the Games–Howell test statistic, df are pair’s own degrees of freedom. The *p*-value in the Games–Howell test is the probability of observing a mean difference between two groups as extreme as (or more extreme than) the one actually found, assuming that the true means of the groups are equal. For the Games–Howell test, only the results for the most relevant pairwise comparisons are presented.

As shown in Table 10, the mean color differences ΔE of grades 2 and 3 are not significantly different. One possible explanation is the subjectivity of visual grading. During visual inspection, each test sample is compared with the reference GP sample. Samples that match the reference are assigned to grade 1, whereas those that deviate are classified as grade 2 or 3 based on the inspector’s subjective assessment of the degree of deviation. Another possible explanation is the absence of an upper color difference ΔE limit for grade 3, which may contribute to greater within-group variance. Greater within-group variance increases the standard error, thereby reducing the Games–Howell test statistic and increasing the associated *p*-value for a given difference between group means.

Since color difference ΔE is the only characteristic that incorporates color information from both the reference and test GP samples, it is selected as the sole characteristic for evaluating different color grades to simplify the analysis. The statistical properties of the color difference ΔE for each grade, as determined by visual inspection, are summarized in Table 11.

**Figure 14:**
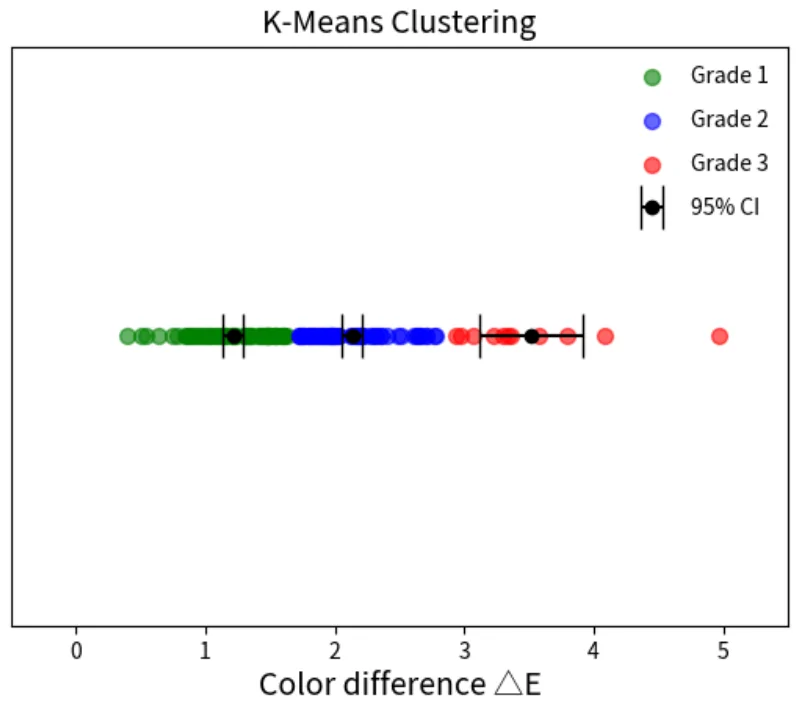
The clustering result of the K-Means clustering algorithm. The 95% confidence intervals (CIs) for each clustering are shown as black dots with error bars.

**Figure 15:**
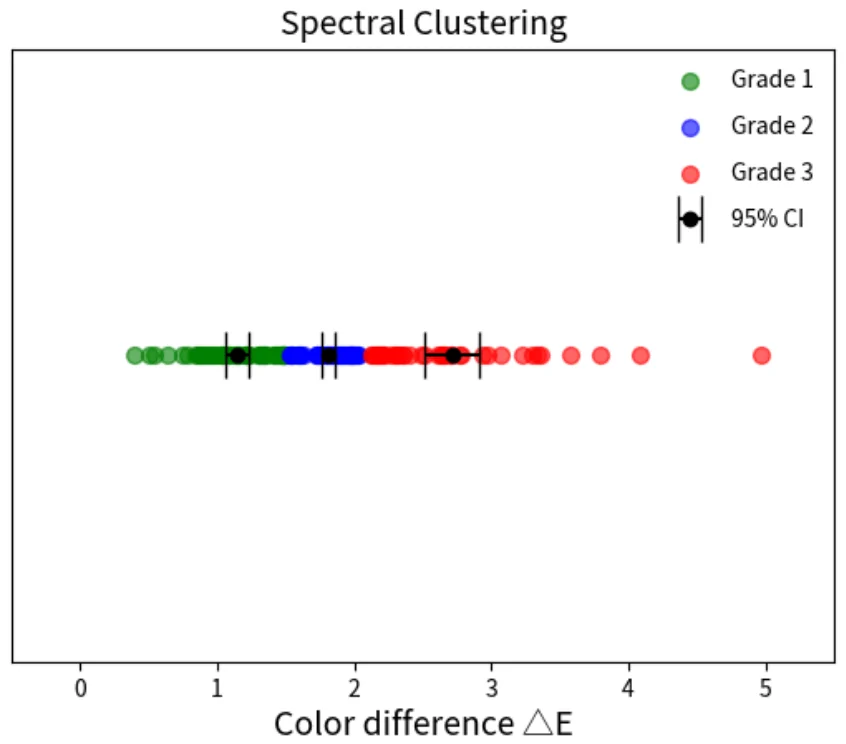
The clustering result of the spectral clustering algorithm. The 95% confidence intervals (CIs) for each clustering are shown as black dots with error bars.

**Figure 16:**
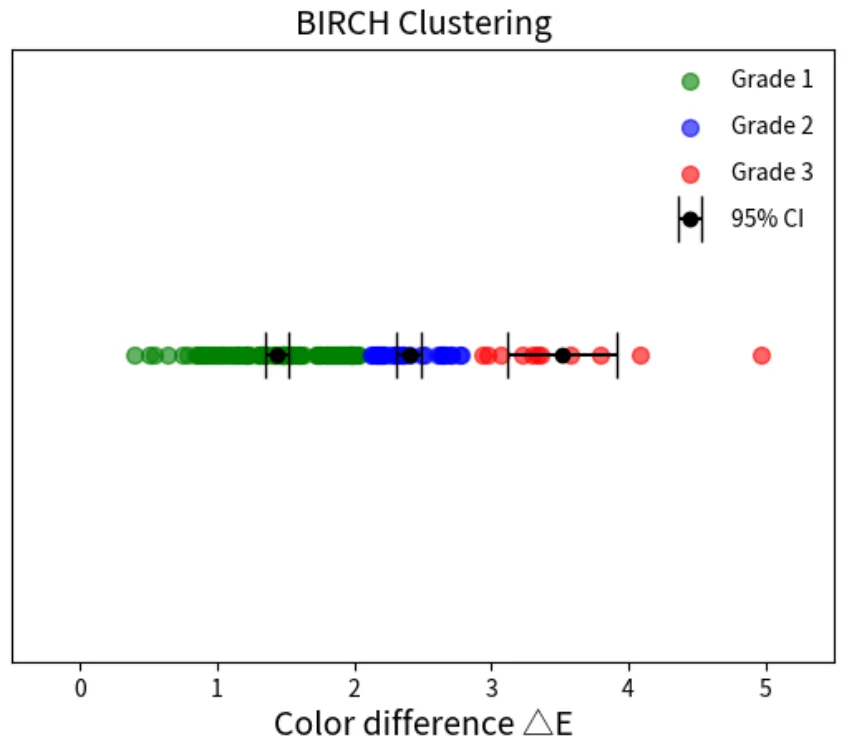
The clustering result of the BIRCH clustering algorithm. The 95% confidence intervals (CIs) for each clustering are shown as black dots with error bars.

**Figure 17:**
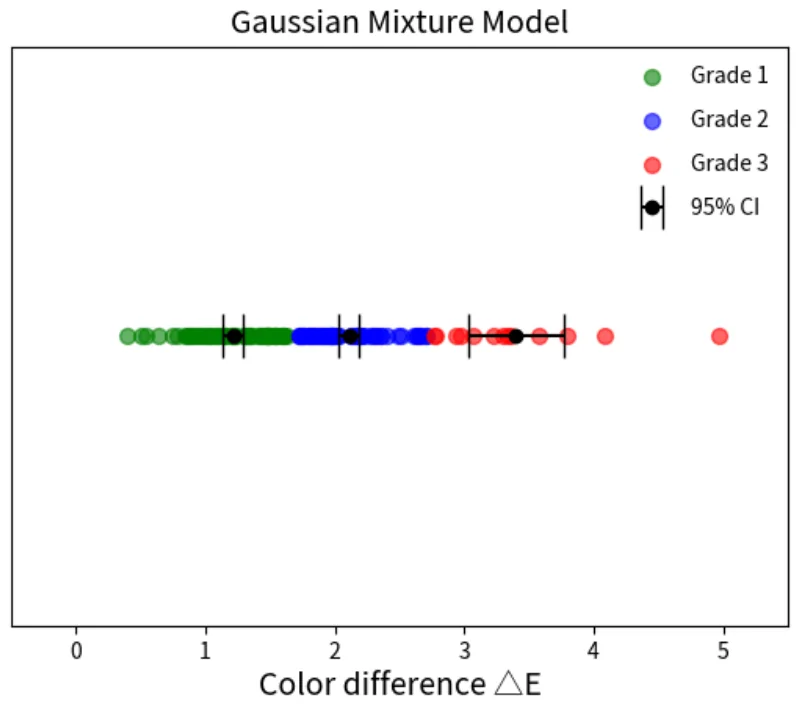
The clustering result of the Gaussian mixture model algorithm. The 95% confidence intervals (CIs) for each clustering are shown as black dots with error bars.

**Figure 18:**
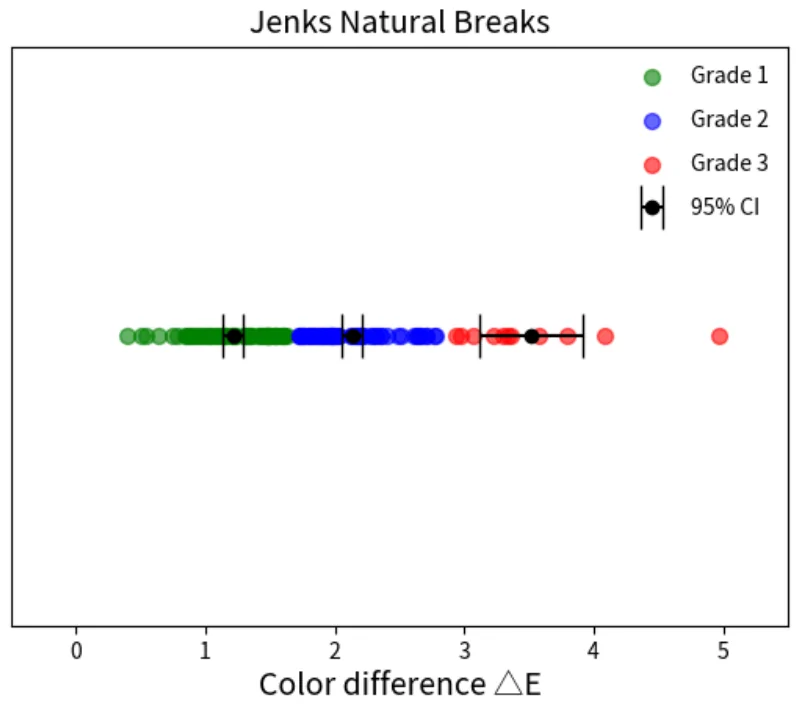
The clustering result of the Jenks natural breaks algorithm. The 95% confidence intervals (CIs) for each clustering are shown as black dots with error bars.

Because the color difference ΔE distributions of different grades are not completely separated, unsupervised machine learning is employed to further optimize the grading based on color difference ΔE. Representative clustering algorithms implemented in the scikit-learn library [60] that allow the number of clusters to be specified are evaluated and compared, including K-Means, spectral clustering, BIRCH, and Gaussian mixture models. Although these clustering algorithms can be used in more complex scenarios, there are no limitations to applying these methods in one-dimensional clustering. In addition, Jenks natural breaks, a simple one-dimensional classification method, is used for comparison. The measured color difference ΔE values are used as the input feature for the unsupervised clustering models:

- K-Means algorithm: When applying the **KMeans** function in scikit-learn, three parameters are specified, while the remaining parameters are retained at their default values. The **n_clusters** parameter is set to 3 to partition the color measurement data into three clusters corresponding to the three visual quality grades in the dataset. The **max_iter** parameter, which specifies the maximum number of iterations for a single run, is set to 100, as larger values do not affect the final clustering results. The **n_init** parameter, which specifies the number of runs with different centroid initializations, is set to 100. Among these runs, the solution with the lowest inertia, i.e., the minimum within-cluster sum of squares (WCSS), is selected. Increasing **n_init** beyond 100 does not produce noticeable changes in the clustering results. Figure 14 presents the clustering results obtained using K-Means.
- Spectral clustering algorithm: When applying the **SpectralClustering** function in scikit-learn, several parameters are specified to obtain an appropriate clustering result, while the remaining parameters are retained at their default values. The parameter **n_clusters = 3** specifies that the data are partitioned into three clusters. The parameter **affinity = ‘nearest_neighbors’** indicates that the affinity matrix is constructed using a nearest-neighbor graph, while **n_neighbors = 10** specifies the number of nearest neighbors used to construct this graph. The parameter **n_init = 100** specifies the number of K-Means initializations used during the label assignment stage, with the solution having the lowest inertia selected. Finally, **assign_labels = ‘kmeans’** indicates that K-Means is employed to assign the final cluster labels in the spectral embedding space. The clustering result obtained using spectral clustering is shown in Figure 15.
- BIRCH: When applying the **Birch** function in scikit-learn, the number of clusters is set to **n_clusters = 3**, and the maximum radius of each sub-cluster is controlled by setting **threshold = 0.01**. The remaining parameters are retained at their default values. Within scikit-learn, **Birch** uses **AgglomerativeClustering** in which **n_clusters** is applied to the centroids of the leaves. Figure 16 shows the clustering result obtained using BIRCH.
- Gaussian mixture model: The following parameters are specified when implementing the **GaussianMixture** function in scikit-learn. **n_components = 3** defines the number of Gaussian components, corresponding to the three clusters to be estimated. **covariance_type = ‘spherical’** assumes that each component has a single variance shared across all feature dimensions. **n_init = 100** specifies that the model is fitted using 100 different initializations, and the solution with the highest probability is retained. Figure 17 shows the clustering result obtained using a Gaussian mixture model.
- Jenks natural breaks: When applying Jenks natural breaks, the number of distinct groups is set to **n_classes = 3**. Figure 18 shows the clustering result using Jenks natural breaks.

The statistical properties of the color difference values for each grade, as determined by different clustering algorithms, are compared in Table 12.

Table 13 presents the confusion matrix for each clustering algorithm, comparing the clustering (predicted) labels against the reference (visual inspection) labels.

The performance of each clustering algorithm is evaluated using three metrics: the adjusted Rand index (ARI) [61], normalized mutual information (NMI) [62,63], and silhouette score [64]. A limitation of the ARI and NMI metrics is that they require ground-truth labels. Although visual inspection results are subjective, they still provide some level of meaningful reference. Therefore, they are used as reference labels to evaluate clustering performance:

- Adjusted Rand Index (ARI): The Rand Index (RI) measures the similarity between two clustering assignments by considering all pairs of samples and determining whether each pair is assigned to the same or different clusters in both the predicted and reference clusterings. The raw RI is then corrected for agreement occurring by chance, resulting in the adjusted Rand index, which is defined by the following formula:
  (41) ARI=∑ijnij2−∑iai2∑jbj2/N212∑iai2+∑jbj2−∑iai2∑jbj2/N2
  where *N* is the total number of samples, $n_{ij}$ is the contingency table cell counts, $a_{i}$ is the sum of elements in row *i* of the contingency table, and $b_{j}$ is the sum of elements in column *j* of the contingency table. The visual inspection results are used as the reference labels for evaluating the clustering performance. An ARI value of 1 indicates perfect agreement between the clustering results and the reference labels, whereas a value close to 0 indicates an agreement comparable to that expected by random chance.
- Normalized Mutual Information (NMI): NMI is a normalized form of mutual information that quantifies the similarity between two clustering assignments on a scale from 0 to 1, where 0 indicates no mutual information and 1 represents perfect agreement. For two clustering assignments *U* and *V*, NMI is defined as:
  (42) $$\mathrm{NMI}\left(U,V\right)=\frac{I(U;V)}{(H(U)+H(V))/2}$$
  (43) $$I\left(U;V\right)=\sum_{i,j}p\left(u_{i},v_{j}\right)\log\left(\frac{p(u_{i},v_{j})}{p\left(u_{i}\right)p\left(v_{j}\right)}\right)$$
  (44) $$H\left(U\right)=-\sum_{i}p\left(u_{i}\right)\;\log p\left(u_{i}\right)$$
  (45) $$H\left(V\right)=-\sum_{j}p\left(v_{j}\right)\;\log p\left(v_{j}\right)$$
  where $p(x_{i})$ is the probability of outcome $x_{i}$, I(U;V) is the mutual information between *U* and *V*, H(U) and H(V) are the entropies of *U* and *V*. The visual inspection results are used as the reference labels in Equation (42).
- Silhouette Score (SS): The silhouette score evaluates clustering quality by measuring how well each data point is matched to its assigned cluster relative to neighboring clusters. For each data point *i*, the silhouette coefficient is calculated as:
  (46) $$s_{i}=\frac{b_{i}-a_{i}}{\max(a_{i},b_{i})}$$
  where $a_{i}$ is the average distance between point *i* and all other points in the same cluster and $b_{i}$ is the average distance between the data point *i* and all points in the nearest neighbouring cluster. The overall silhouette score is the mean over all *n* samples:
  (47) $$SS=\frac{1}{n}\sum_{i=1}^{n}s_{i}$$
  The silhouette score ranges from −1 to 1. A value close to 1 indicates compact and well-separated clusters, whereas a value near 0 suggests overlapping or poorly separated clusters. A negative value indicates that some data points may have been assigned to inappropriate clusters.

Table 14 summarizes the results of the three evaluation metrics for each clustering algorithm considered in this study. BIRCH clustering achieves the highest ARI and NMI scores, whereas K-Means clustering yields the highest silhouette score among the evaluated algorithms. Overall, the color difference ΔE cutoff thresholds derived from BIRCH clustering best align with the manufacturer’s practical color quality control requirements.

Bootstrap resampling is used to assess the stability of the clustering results. First, a reference clustering model is fitted to the complete dataset. Subsequently, 1000 bootstrap datasets are generated by randomly sampling observations with replacement, with each dataset containing the same number of observations as the original dataset. Consequently, individual observations could appear multiple times or be omitted from a bootstrap dataset.

For each bootstrap dataset, clustering algorithm is refitted using the same settings as the reference model. Each fitted model is then used to assign cluster labels to all observations in the original dataset. Agreement between these assignments and the reference clustering is quantified using the adjusted Rand index (ARI), which is unaffected by permutations of cluster labels. The mean and standard deviation of the bootstrap ARI scores are calculated to summarize clustering stability. The results are shown in Table 15. Higher ARI values indicate greater consistency with the original clustering under resampling.

The same device and color measurement procedure are also applied to GP samples with different specifications, including variations in dye concentration and color. The results demonstrate that the proposed device remains capable of detecting subtle color variations across these different GP specifications. However, detailed results are not presented in this article due to commercial confidentiality considerations.

Our device may be utilized for the analysis of a wide range of polymer additives if a similar color measurement mechanism works. These additives play critical roles in both polymer processing and the enhancement of material performance. Color analysis offers a valuable approach for evaluating formulation changes and optimizing the properties of plastic products. For instance, the clarification of polymer materials is an important concern in the plastics industry because clarifying agents influence the polymer microstructure and promote crystallization. A more compact crystalline structure enhances transparency and improves the visual appearance of the material. Since variations in crystal morphology affect light transmission characteristics, color measurement instruments can effectively monitor the crystallization process by quantifying changes in transmitted light.

Fillers are another commonly used class of additives in polymer manufacturing, primarily employed to reduce production costs and increase material yield. However, fillers can also significantly influence the color characteristics of the final product. Continuous monitoring of color changes throughout product development enables manufacturers to optimize the concentration of colorants and other additives during processing, thereby improving color quality and minimizing material waste.

In addition to evaluating colorants, color measurement techniques can provide insight into molecular and structural changes associated with stabilizers, fillers, processing aids, and antimicrobial additives. Our color measurement system is capable of detecting subtle formulation variations, enabling a more efficient and sensitive evaluation methodology. The proposed device and analytical approach can help ensure the stability, uniformity, and consistency of plastic products while reducing material waste.

## 5. Conclusions

This study presents a practical approach to addressing the limitations of conventional color-grading methods currently used in the plastics industry. A transmission-based color measurement system is proposed for detecting subtle color variations in large-size and translucent GP samples. Compared with a conventional commercial colorimeter, the proposed system demonstrates superior discrimination capability for distinguishing among different color grades of GP samples. Welch’s ANOVA and Games–Howell test are performed to determine whether the mean values of the measured color characteristics differ significantly among different grades. Among the evaluated characteristics, the color difference ΔE is the only characteristic that contains color information from both the reference and test GP samples and is therefore selected as the sole characteristic for subsequent color-grade evaluation. Unsupervised machine learning is then employed to further optimize the grading based on color difference ΔE. Five clustering algorithms, namely K-Means, spectral clustering, BIRCH, a Gaussian mixture model, and Jenks natural breaks, are evaluated and compared. Among these methods, BIRCH clustering achieved the highest ARI (0.50) and NMI (0.35) scores, whereas K-Means yielded the highest silhouette score (0.57). Overall, the color difference cutoff thresholds derived from BIRCH clustering most effectively satisfy the practical requirements of color quality control in the manufacturing process. The ability to achieve reliable discrimination of subtle color variations using a low-cost and effective system represents a promising approach to polymer characterization and industrial quality control. Overall, this work establishes a practical foundation for objective and quantitative color grading, AI-assisted quality assurance, and the further development of smart manufacturing in the plastics industry.

## Figures and Tables

**Table 1:** Comparisons of the performance between $\Delta E_{ab}^{*}$ and $\Delta E_{00}^{*}$.

Sample	Grade	$\Delta E_{\mathrm{ab}}^{*}$	$\Delta E_{00}^{*}$
1	1	0.57 ± 0.08	0.55 ± 0.08
2	1	1.17 ± 0.14	0.98 ± 0.12
3	1	1.35 ± 0.18	1.14 ± 0.15
4	2	1.79 ± 0.20	1.31 ± 0.14
5	2	1.91 ± 0.19	1.48 ± 0.15
6	2	2.09 ± 0.23	1.63 ± 0.18
7	3	2.70 ± 0.27	1.96 ± 0.20
8	3	2.84 ± 0.26	2.07 ± 0.19
9	3	3.06 ± 0.31	2.28 ± 0.23

**Table 2:** Summary of the repeated measurements. L* is lightness, a* is the red–green axis value, b* is the yellow–blue axis value, C is the chroma value, and h is the hue.

	L*	a*	b*	C	h
Mean	32.26	3.65	−10.23	10.86	289.60
Standard deviation	0.32	0.04	0.15	0.14	0.30
Coefficient of variation	1.0 %	1.2 %	1.4 %	1.3 %	0.1 %

**Table 3:** Comparison of the measurements between our device and a commercial colorimeter with the same set of GP samples. ΔE is the color difference.

Sample	Grade	Our Device ΔE	Colorimeter ΔE
1	1	0.57 ± 0.08	0.25 ± 0.04
2	1	1.17 ± 0.14	0.44 ± 0.05
3	1	1.35 ± 0.18	0.33 ± 0.04
4	2	1.79 ± 0.20	0.43 ± 0.04
5	2	1.91 ± 0.19	0.58 ± 0.07
6	2	2.09 ± 0.23	0.55 ± 0.08
7	3	2.70 ± 0.27	0.59 ± 0.08
8	3	2.84 ± 0.26	0.66 ± 0.09
9	3	3.06 ± 0.31	0.70 ± 0.08

**Table 4:** Summary of color difference ΔE separability between grades 1 and 2 ($S_{12}$), grades 1 and 3 ($S_{13}$), and grades 2 and 3 ($S_{23}$).

	$S_{12}$	$S_{13}$	$S_{23}$
Separability	14.9	16.5	2.1

**Table 5:** Summary, Welch’s ANOVA and Games–Howell test of lightness L*.

Summary			
**Groups**	**Count**	**Average**	**Variance**
Grade 3R	5	34.18	0.81
Grade 2R	2	33.81	0.15
Grade 1	100	33.34	0.29
Grade 2L	7	32.54	1.15
Grade 3L	14	32.46	0.66
Welch’s ANOVA			
**F-Statistic**	${\mathrm{df}}_{1}$	${\mathrm{df}}_{2}$	***p*** **-Value**
5.01	4.00	5.70	0.044
Games–Howell Pairwise Comparisons			
**Groups**	**Statistic**	df	***p*** **-Value**
Grade 1 vs. grade 2R	−1.71	1.08	0.322
Grade 1 vs. grade 2L	1.94	6.24	0.099
Grade 1 vs. grade 3R	−2.07	4.15	0.104
Grade 1 vs. grade 3L	3.90	14.64	0.001
Grade 2R vs. grade 3R	−0.76	4.64	0.484
Grade 2L vs. grade 3L	0.17	9.57	0.867

**Table 6:** Summary, Welch’s ANOVA and Games–Howell test of red–green axis value a*.

Summary			
**Groups**	**Count**	**Average**	**Variance**
Grade 3R	4	4.23	0.01
Grade 2R	5	3.94	0.06
Grade 1	100	3.36	0.04
Grade 2L	4	3.03	0.03
Grade 3L	15	2.93	0.04
Welch’s ANOVA			
**F-Statistic**	${\mathrm{df}}_{1}$	${\mathrm{df}}_{2}$	***p*** **-Value**
81.76	4.00	9.19	<0.001
Games–Howell Pairwise Comparisons			
**Groups**	**Statistic**	df	***p*** **-Value**
Grade 1 vs. grade 2R	−5.13	4.26	0.006
Grade 1 vs. grade 2L	3.62	3.30	0.031
Grade 1 vs. grade 3R	−16.04	3.98	<0.001
Grade 1 vs. grade 3L	7.80	15.53	<0.001
Grade 2R vs. grade 3R	−2.39	5.51	0.058
Grade 2L vs. grade 3L	0.88	5.09	0.420

**Table 7:** Summary, Welch’s ANOVA and Games–Howell test of yellow–blue axis value b*.

Summary			
**Groups**	**Count**	**Average**	**Variance**
Grade 3R	15	−7.31	0.46
Grade 2R	5	−8.06	1.12
Grade 1	100	−9.66	0.46
Grade 2L	4	−11.15	0.11
Grade 3L	4	−12.12	0.06
Welch’s ANOVA			
**F-Statistic**	${\mathrm{df}}_{1}$	${\mathrm{df}}_{2}$	***p*** **-Value**
126.79	4.00	10.02	<0.001
Games–Howell Pairwise Comparisons			
**Groups**	**Statistic**	** df **	***p*** **-Value**
Grade 1 vs. grade 2R	−3.34	4.16	0.027
Grade 1 vs. grade 2L	8.41	4.10	<0.001
Grade 1 vs. grade 3R	−12.54	18.43	<0.001
Grade 1 vs. grade 3L	17.14	4.94	<0.001
Grade 2R vs. grade 3R	−1.50	5.14	0.192
Grade 2L vs. grade 3L	4.67	5.64	0.004

**Table 8:** Summary, Welch’s ANOVA and Games–Howell test of chroma value C.

Summary			
**Groups**	**Count**	**Average**	**Variance**
Grade 3R	4	12.83	0.07
Grade 2R	4	11.85	0.14
Grade 1	100	10.22	0.45
Grade 2L	5	8.65	1.17
Grade 3L	15	7.87	0.43
Welch’s ANOVA			
**F-Statistic**	${\mathrm{df}}_{1}$	${\mathrm{df}}_{2}$	***p*** **-Value**
133.29	4.00	9.82	<0.001
Games–Howell Pairwise Comparisons			
**Groups**	**Statistic**	df	***p*** **-Value**
Grade 1 vs. grade 2R	−8.25	3.83	0.001
Grade 1 vs. grade 2L	3.22	4.15	0.031
Grade 1 vs. grade 3R	−17.76	4.76	<0.001
Grade 1 vs. grade 3L	12.88	18.64	<0.001
Grade 2R vs. grade 3R	−4.34	5.40	0.006
Grade 2L vs. grade 3L	1.51	5.02	0.191

**Table 9:** Summary, Welch’s ANOVA and Games–Howell test of hue h.

Summary			
**Groups**	**Count**	**Average**	**Variance**
Grade 3R	16	291.85	3.17
Grade 2R	9	290.62	1.47
Grade 1	100	289.17	1.54
Grade 2L	0	-	-
Grade 3L	3	288.63	0.22
Welch’s ANOVA			
**F-Statistic**	${\mathrm{df}}_{1}$	${\mathrm{df}}_{2}$	***p*** **-Value**
14.72	3.00	9.43	<0.001
Games–Howell Pairwise Comparisons			
**Groups**	**Statistic**	df	***p*** **-Value**
Grade 1 vs. grade 2R	−3.43	9.56	0.007
Grade 1 vs. grade 2L	-	-	-
Grade 1 vs. grade 3R	−5.80	17.40	<0.001
Grade 1 vs. grade 3L	1.79	2.91	0.174
Grade 2R vs. grade 3R	−2.04	21.94	0.054
Grade 2L vs. grade 3L	-	-	-

**Table 10:** Summary, Welch’s ANOVA and Games–Howell test of color difference ΔE.

Summary			
**Groups**	**Count**	**Average**	**Variance**
Grade 3	19	2.93	0.60
Grade 2	9	2.55	0.27
Grade 1	100	1.55	0.25
Welch’s ANOVA			
**F-Statistic**	${\mathrm{df}}_{1}$	${\mathrm{df}}_{2}$	***p*** **-Value**
39.03	2.00	17.11	<0.001
Games–Howell Pairwise Comparisons			
**Groups**	**Statistic**	df	***p*** **-Value**
Grade 1 vs. grade 2	−5.59	9.41	<0.001
Grade 1 vs. grade 3	−7.46	20.96	<0.001
Grade 2 vs. grade 3	−1.52	22.69	0.142

**Table 11:** The statistical properties of the color difference ΔE, including count, mean value, 95% confidence interval (CI), minimum and maximum values for each grade based on visual inspection.

Visual Inspection	Grade	Count	Mean	95% CI	(Min, Max)
	1	100	1.55	1.45–1.65	(0.39, 2.77)
	2	9	2.55	2.16–2.95	(1.94, 3.33)
	3	19	2.93	2.56–3.30	(1.99, 4.97)

**Table 12:** The statistical properties of the color difference ΔE, including count, mean value, 95% confidence interval (CI), minimum and maximum values for each grade based on clustering results.

**K-Means Clustering**	**Grade**	**Count**	**Mean**	**95% CI**	**(Min, Max)**
	1	59	1.21	1.13–1.29	(0.39, 1.63)
	2	58	2.13	2.05–2.21	(1.72, 2.78)
	3	11	3.51	3.11–3.91	(2.94, 4.97)
**Spectral Clustering**	**Grade**	**Count**	**Mean**	**95% CI**	**(Min, Max)**
	1	50	1.15	1.07–1.23	(0.39, 1.50)
	2	39	1.81	1.76–1.86	(1.53, 2.03)
	3	39	2.71	2.51–2.91	(2.12, 4.97)
**BIRCH Clustering**	**Grade**	**Count**	**Mean**	**95% CI**	**(Min, Max)**
	1	89	1.44	1.35–1.52	(0.39, 2.03)
	2	28	2.40	2.31–2.49	(2.12, 2.78)
	3	11	3.51	3.11–3.91	(2.94, 4.97)
**GMM Clustering**	**Grade**	**Count**	**Mean**	**95% CI**	**(Min, Max)**
	1	59	1.21	1.13–1.29	(0.39, 1.63)
	2	56	2.11	2.03–2.19	(1.72, 2.70)
	3	13	3.40	3.03–3.77	(2.77, 4.97)
**Jenks Natural Breaks**	**Grade**	**Count**	**Mean**	**95% CI**	**(Min, Max)**
	1	59	1.21	1.13–1.29	(0.39, 1.63)
	2	58	2.13	2.05–2.21	(1.72, 2.78)
	3	11	3.51	3.11–3.91	(2.94, 4.97)

**Table 13:** Confusion matrix of clustering results.

**K-Means Clustering**	**Clustering Grade 1**	**Clustering Grade 2**	**Clustering Grade 3**
Reference grade 1	59	41	0
Reference grade 2	0	7	2
Reference grade 3	0	10	9
**Spectral Clustering**	**Clustering Grade 1**	**Clustering Grade 2**	**Clustering Grade 3**
Reference grade 1	50	35	15
Reference grade 2	0	2	7
Reference grade 3	0	2	17
**BIRCH Clustering**	**Clustering Grade 1**	**Clustering Grade 2**	**Clustering Grade 3**
Reference grade 1	85	15	0
Reference grade 2	2	5	2
Reference grade 3	2	8	9
**GMM Clustering**	**Clustering Grade 1**	**Clustering Grade 2**	**Clustering Grade 3**
Reference grade 1	59	40	1
Reference grade 2	0	7	2
Reference grade 3	0	9	10
**Jenks Natural Breaks**	**Clustering Grade 1**	**Clustering Grade 2**	**Clustering Grade 3**
Reference grade 1	59	41	0
Reference grade 2	0	7	2
Reference grade 3	0	10	9

**Table 14:** Clustering results evaluated by adjusted Rand index (ARI), normalized mutual information (NMI), and silhouette score (SS) metrics.

	K-Means Clustering	Spectral Clustering	BIRCH Clustering	GMM Clustering	Jenks Natural Breaks
ARI	0.22	0.17	0.50	0.23	0.22
NMI	0.32	0.26	0.35	0.30	0.32
SS	0.57	0.43	0.47	0.56	0.57

**Table 15:** The mean and standard deviation of the bootstrap ARI scores for assessing clustering stability.

	Mean	Standard Deviation
K-Means clustering	0.84	0.18
Spectral clustering	0.69	0.17
BIRCH clustering	0.71	0.28
GMM clustering	0.62	0.26
Jenks natural breaks	0.82	0.18

## Data Availability

The original contributions presented in this study are included in the article. Further inquiries can be directed to the corresponding author.

## Abbreviations

The following abbreviations are used in this manuscript:

AI	Artificial intelligence
AD	Alzheimer’s disease
ANOVA	Analysis of variance
ARI	Adjusted Rand index
BIRCH	Balanced iterative reducing and clustering using hierarchies
CF	Clustering feature
GMM	Gaussian mixture model
GP	General-purpose polystyrene
GUI	Graphical user interface
HSI	Hyperspectral imaging
LED	Light-emitting diode
LCD	Liquid-crystal display
NMI	Normalized mutual information
MSI	Multispectral imaging
RI	Rand index
RGB	Red, green, and blue color values
SCM	Supply chain management
SS	Silhouette score
